# Supplementary figures and images for: Meta-analysis of single-cell RNA sequencing co-expression in human neural organoids reveals their high variability in recapitulating primary tissue
Source: PLoS Biol. 2024 Dec 2;22(12):e3002912. doi: 10.1371/journal.pbio.3002912 (PMC11637388; doi:10.1371/journal.pbio.3002912)

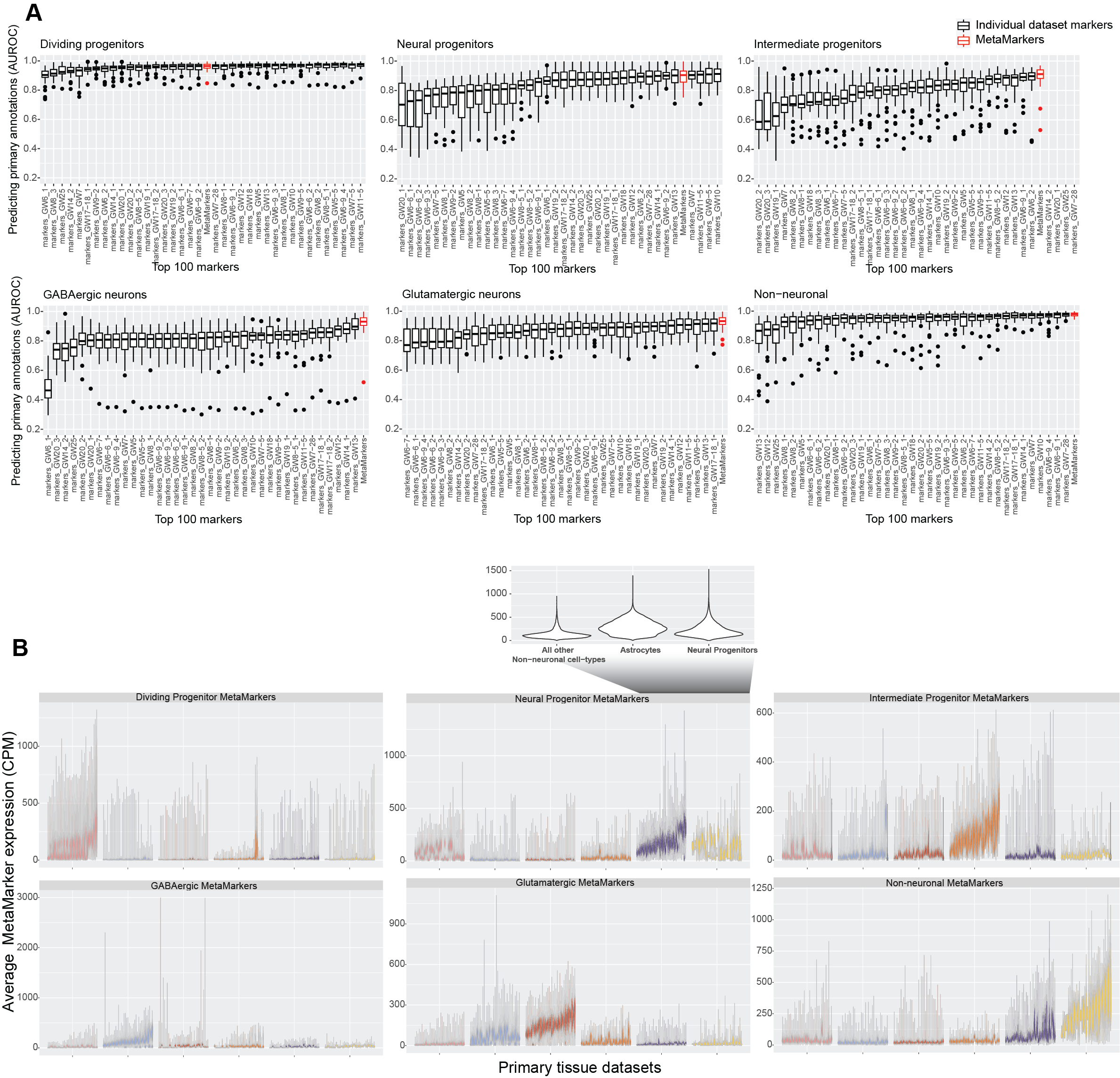

Supplement: S1 Fig — (A) Boxplots of AUROCs for predicting cell type annotations across all primary tissue data sets using the top 100 marker genes per individual primary tissue data set compared to MetaMarkers (red). Data sets are ordered by their median performance, providing the rank distributions in Fig 2D. (B) Distributions of averaged gene expression for the top 100 MetaMarkers across all annotated primary tissue data sets with leave-one-out cross-validation. Fig 2E is the aggregate over these individual data set distributions. Inset displays the average neural progenitor MetaMarker expression for neural progenitor, astrocyte, and all non-astrocyte non-neuronal cells. Underlying data can be found in the Zenodo repository (doi:10.5281/zenodo.13946248). (PNG) [file pbio.3002912.s001.png]

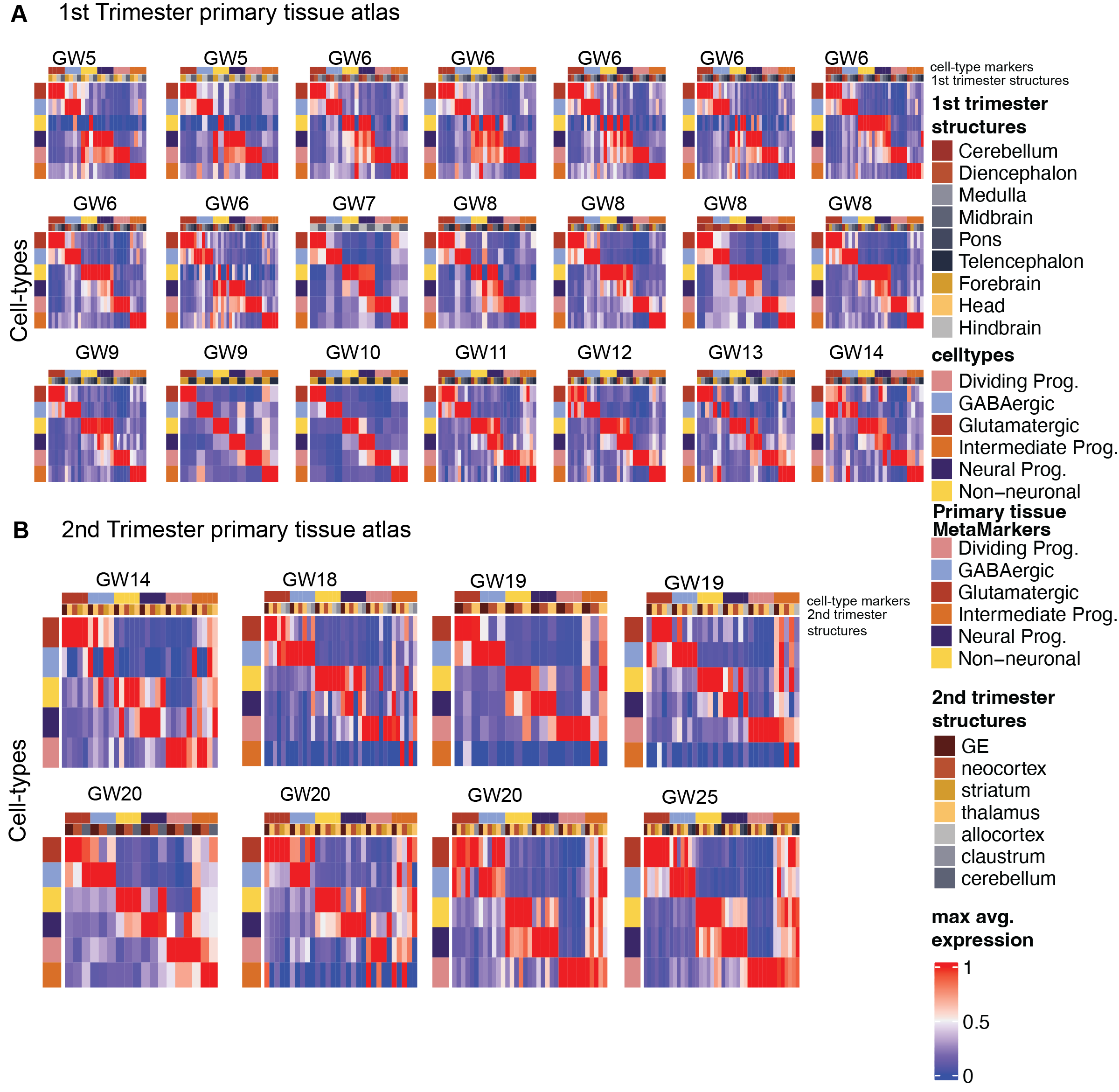

Supplement: S2 Fig — (A) Heatmaps of maximum normalized average MetaMarker expression for cell types and brain regions of the first trimester annotated primary tissue atlas. Cell types comprise the rows with MetaMarker gene expression for cells from each annotated brain region comprising the columns. Data is maximum normalized per region/column. (B) Heatmaps of maximum normalized average MetaMarker expression for cell types and brain regions of the second trimester annotated primary tissue atlas. Cell types comprise the rows with MetaMarker gene expression for cells from each annotated brain region comprising the columns. Data is maximum normalized per region/column. Underlying data can be found in the Zenodo repository (doi:10.5281/zenodo.13946248). (PNG) [file pbio.3002912.s002.png]

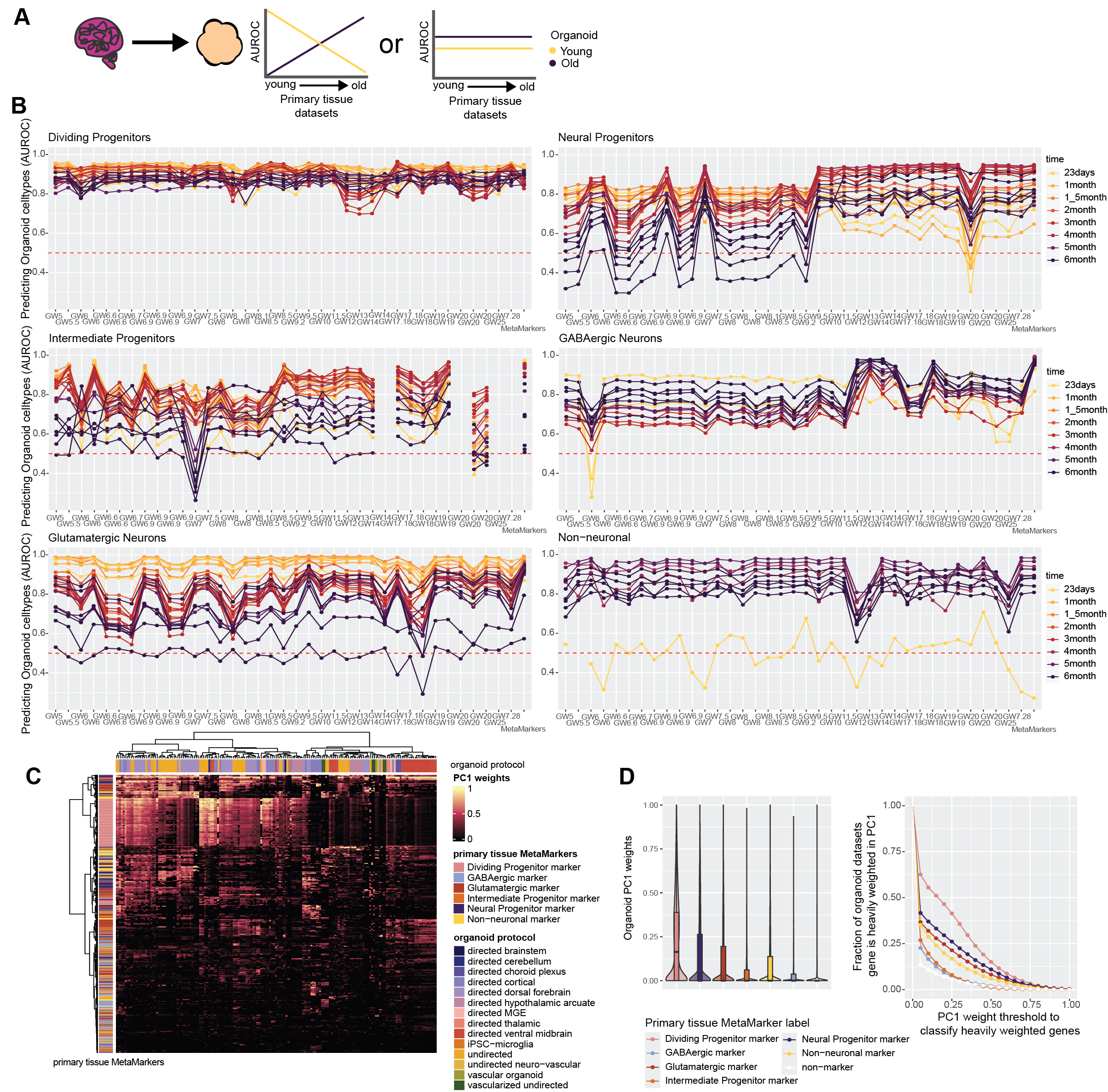

Supplement: S3 Fig — (A) Schematic showing 2 potential outcomes when comparing cell type marker expression between primary tissue and organoid data on a temporal axis. There may be a temporal relationship, with younger organoids recapitulating younger primary tissue marker expression over older primary tissue marker expression and vice versa for older organoids, or there may be no temporal relationship. (B) Line plots showing the cell type prediction AUROCs using top 100 markers from individual primary tissue data sets for all organoid time points. Primary tissue data sets on the x-axis are ordered from youngest to oldest. (C) Heatmap of min-max normalized eigenvalues for primary tissue MetaMarkers within the first principal component of each organoid data set. (D) MetaMarker and non-marker gene set distributions of normalized PC1 eigenvalues across all organoid data sets (left plot). The right plot depicts the fraction of data sets each MetaMarker gene (the average for each MetaMarker gene set is reported) is called as “heavily weighted” in PC1 for a given normalized eigenvalue threshold (x-axis). Underlying data can be found in the Zenodo repository (doi:10.5281/zenodo.13946248). (PNG) [file pbio.3002912.s003.png]

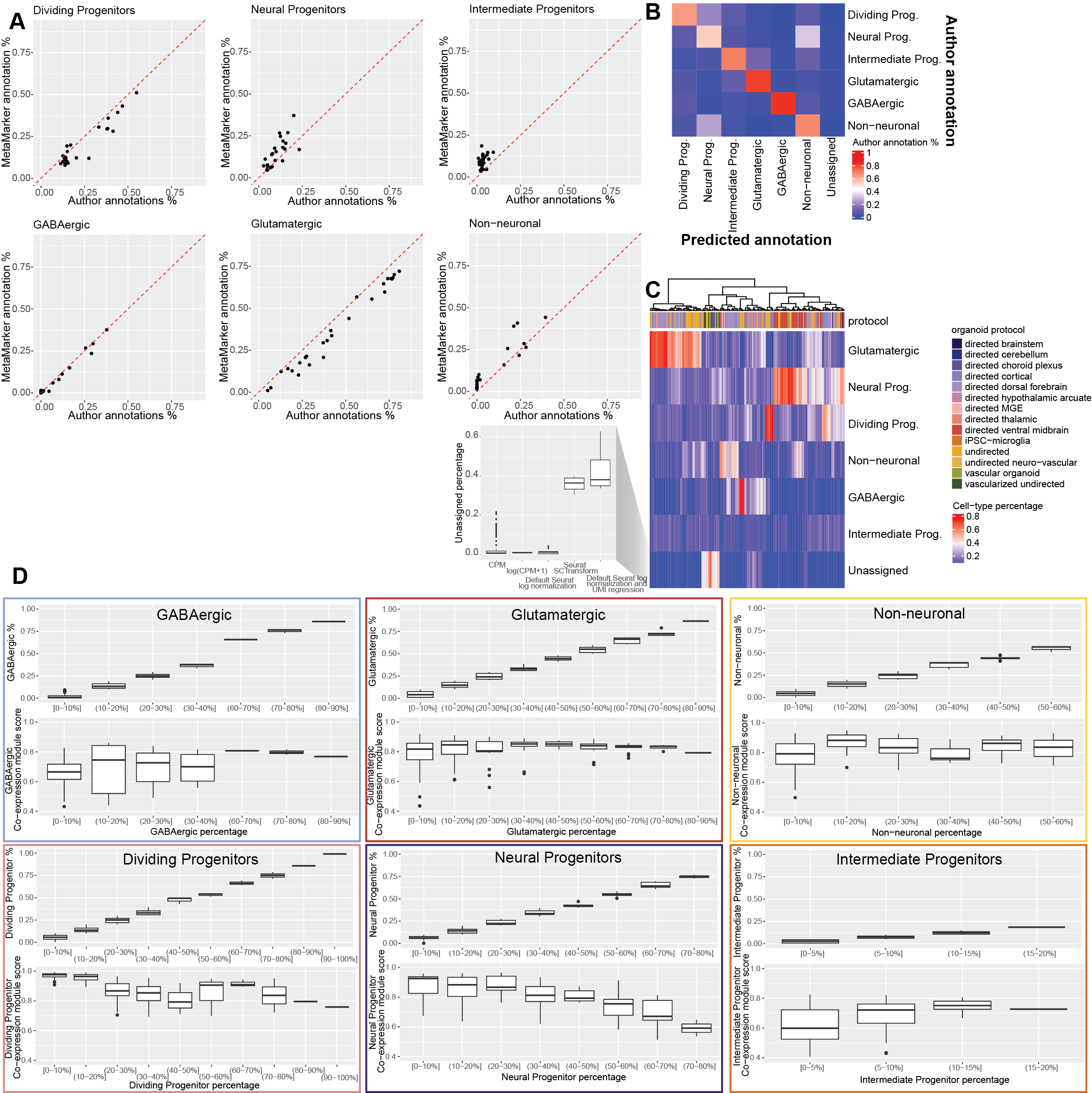

Supplement: S4 Fig — (A) Scatter plots comparing the cell type annotation percentage of individual organoid data sets from the annotated directed dorsal forebrain temporal data sets, with author-provided annotations on the x-axis and annotations determined by MetaMarker expression on the y-axis. (B) Confusion matrix for the results in A, comparing the MetaMarker predicted annotations to the author provided annotations. (C) Heatmap displaying the predicted cell type percentages (rows) of all the organoid data sets (columns), hierarchically clustered by the organoid protocol. The plot to the left of the heatmap depicts the distributions of unassigned cell percentages per data set, where large percentages of unassigned cells are dependent on the expression normalization used in individual studies. Studies with those normalizations were excluded from S4D and S5C Figs. (D) Boxplot distributions comparing the predicted cell type percentage (top boxplot plot per cell type, binned in intervals of 10-percentage points) to the co-expression module score (bottom boxplot plot per cell type) for all neural organoid data sets. The x-axes are the same for the top and bottom sets of boxplots per cell type. Underlying data can be found in the Zenodo repository (doi:10.5281/zenodo.13946248). (PNG) [file pbio.3002912.s004.png]

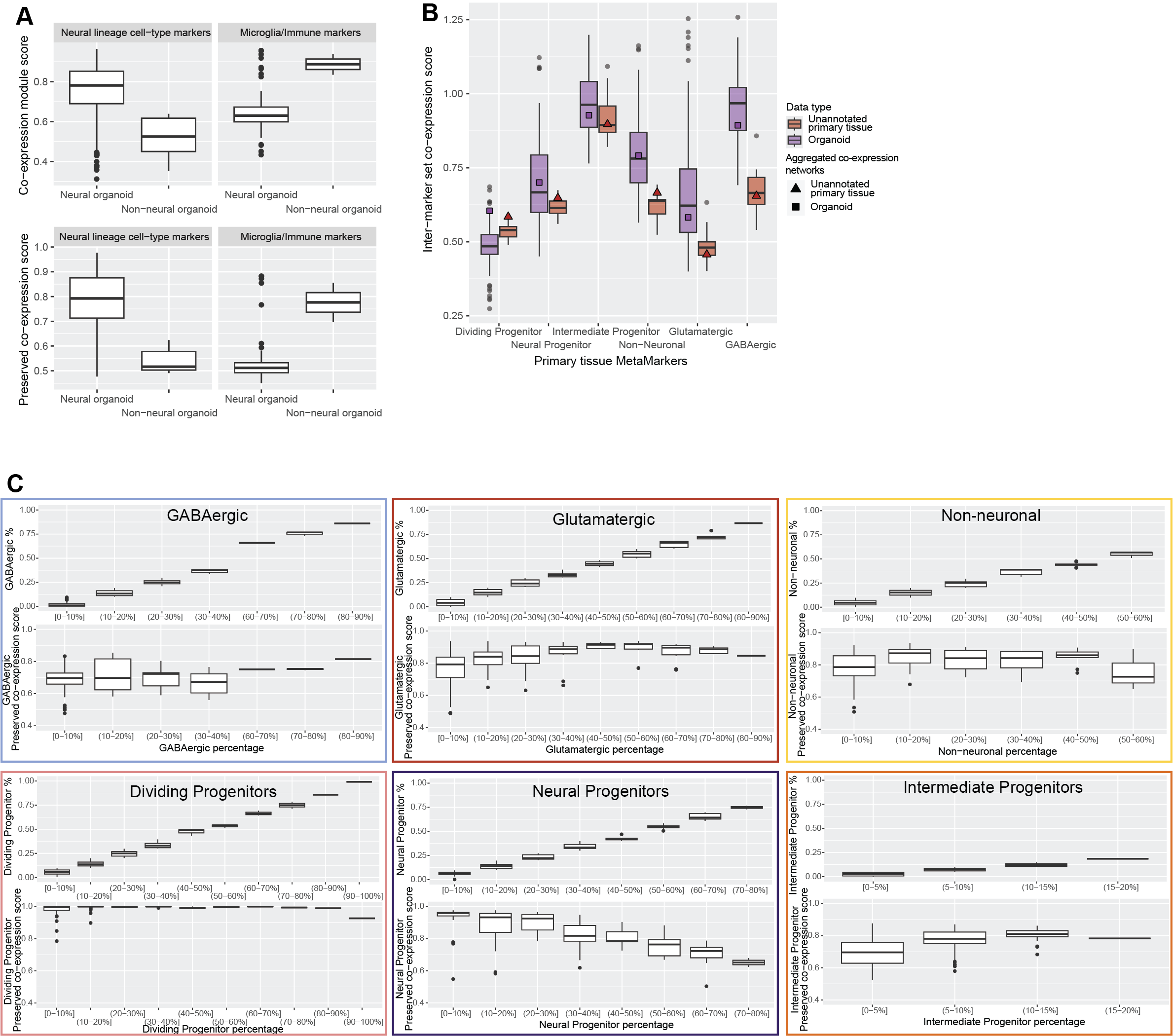

Supplement: S5 Fig — (A) Boxplots comparing the co-expression module scores (top row) for the neural lineage (neural progenitor, intermediate progenitor, glutamatergic, GABAergic, and non-neuronal MetaMarkers) or microglia/immune MetaMarkers between the neural organoid and non-neural organoid data sets. Bottom row of boxplots depicts the preserved co-expression scores. (B) Boxplots depicting the raw inter-marker set co-expression ratios across MetaMarker gene sets for the unannotated primary tissue and neural organoid data sets, standardized ratios are in Fig 3E. Special characters denote the scores of the aggregate co-expression networks. (C) Boxplot distributions comparing the predicted cell type percentage (top boxplot plot per cell type, binned in intervals of 10-percentage points) to the preserved co-expression score (bottom boxplot plot per cell type) for all neural organoid data sets. The x-axes are the same for the top and bottom sets of boxplots per cell type. Underlying data can be found in the Zenodo repository (doi:10.5281/zenodo.13946248). (PNG) [file pbio.3002912.s005.png]

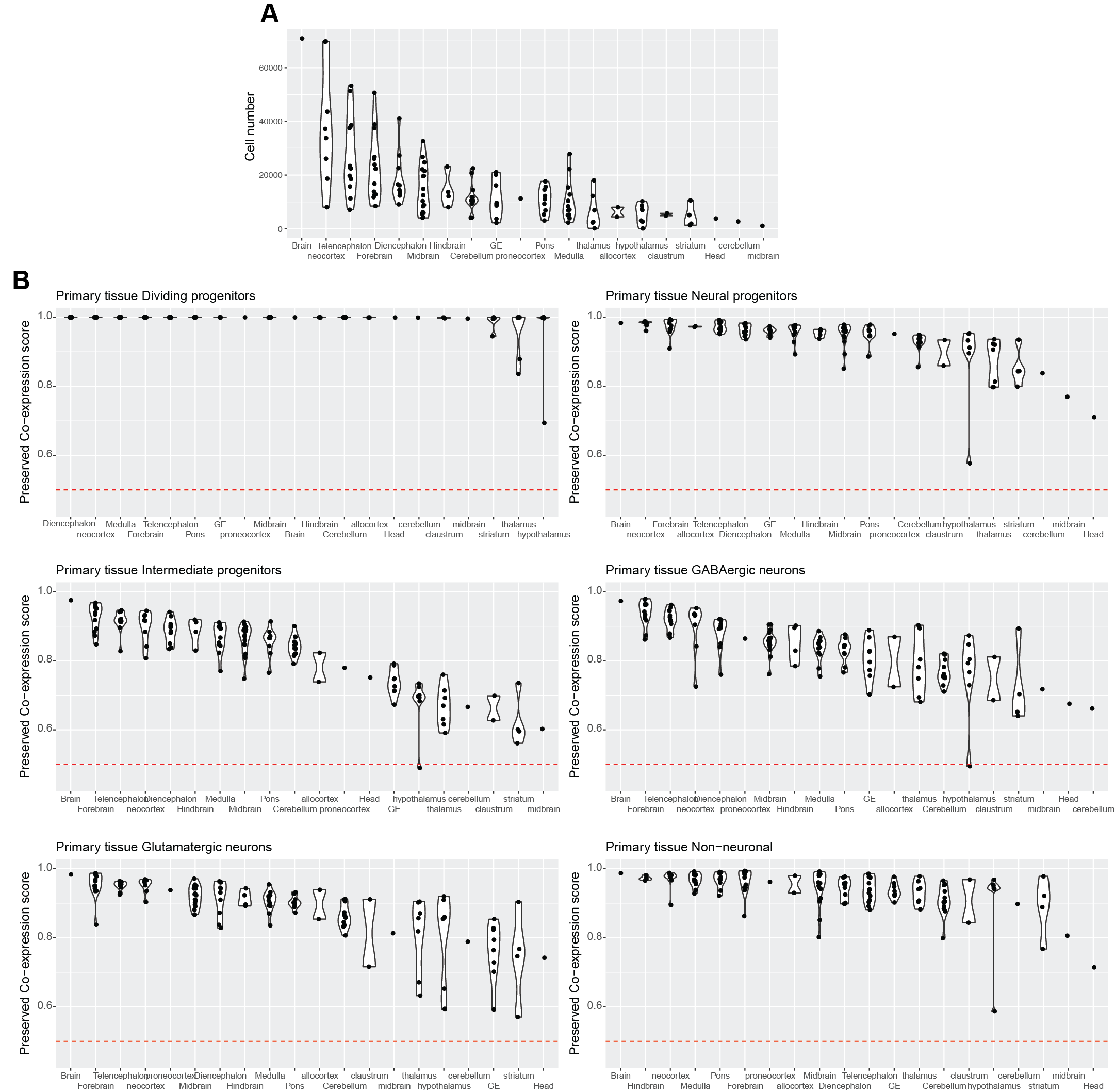

Supplement: S6 Fig — (A) Violin and dotplots displaying the number of sampled cells per annotated brain region across the cross-regional first and second trimester primary tissue data sets. (B) Violin and dotplots displaying the preserved co-expression scores of the top 100 MetaMarkers for each of our 6 cell type annotations, separated by brain region. Underlying data can be found in the Zenodo repository (doi:10.5281/zenodo.13946248). (PNG) [file pbio.3002912.s006.png]

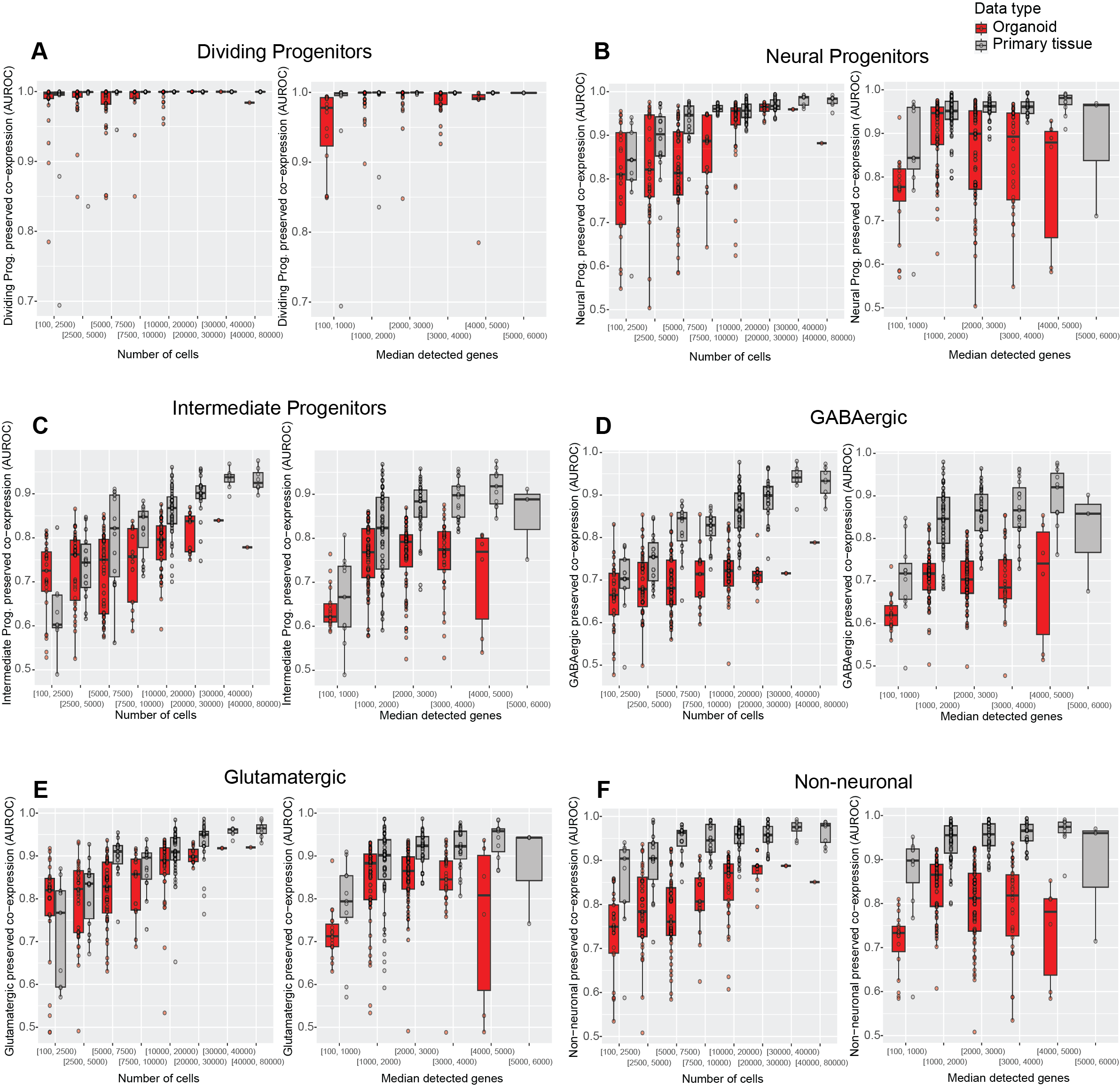

Supplement: S7 Fig — (A) Boxplots and dotplots comparing the preserved co-expression scores for the top 100 dividing progenitor MetaMarkers for all neural organoid data sets (gray) and the region-specific primary tissue data sets (red) from S6 Fig. All data sets are binned on the x-axis by the number of cells present (left-most plot) or the median number of detected genes (right-most plot) per data set. (B) Same as A, but for the top 100 neural progenitor MetaMarkers. (C) Same as A, but for the top 100 intermediate progenitor MetaMarkers. (D) Same as A, but for the top 100 glutamatergic MetaMarkers. (E) Same as A, but for the top 100 GABAergic MetaMarkers. (F) Same as A, but for the top 100 non-neuronal MetaMarkers. Underlying data can be found in the Zenodo repository (doi:10.5281/zenodo.13946248). (PNG) [file pbio.3002912.s007.png]

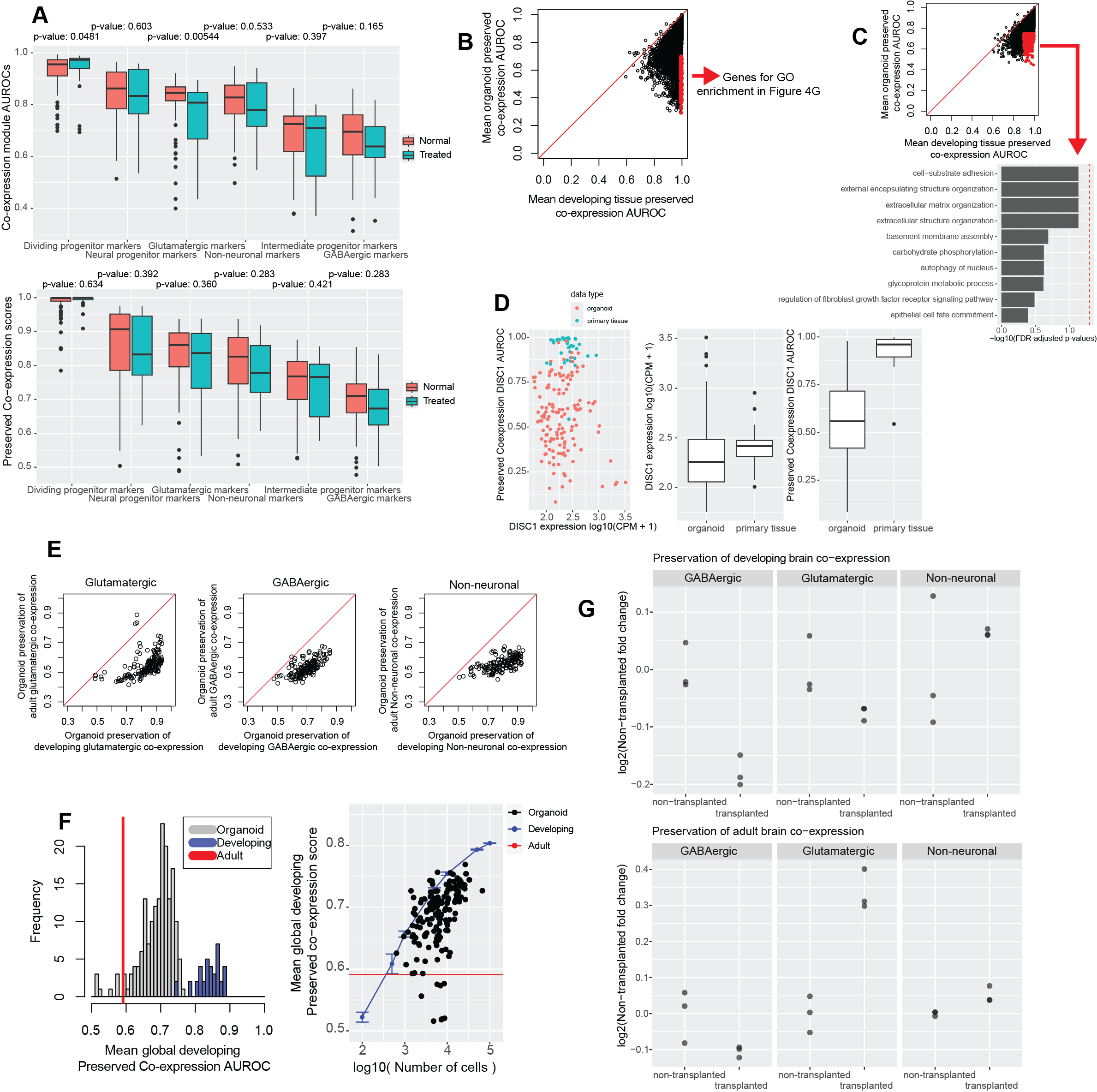

Supplement: S8 Fig — (A) Boxplots comparing either the co-expression module scores or preserved co-expression scores by cell type between normal and treated organoids. (B) Scatter plot showing the average preserved developing brain co-expression AUROC of individual genes, comparing the average across developing brain networks (x-axis) against the average across organoid networks (y-axis). The points colored in red are genes with developing brain scores > = 0.99 and organoid scores < 0.70. (C) Same as in B, only using primary tissue co-expression networks derived using only the 6 broad annotated cell types. Points in red are genes with developing brain scores > = 0.90 and organoid scores < 0.70. The bar plot shows the top 10 GO terms determined by p-value for GO set enrichment of the genes in red. (D) Scatter plot comparing the expression of DISC1 (log10(CPM+1)) to the preserved co-expression score of DISC1 (computed using primary tissue co-expression networks derived from just the neural lineage cell types) for all primary tissue and organoid data sets that used CPM normalization. The boxplots compare DISC1 expression (left) and DISC1 preserved co-expression (right) between primary tissue and neural organoids. (E) Scatterplots showing the preserved co-expression scores of either the top 100 developing brain MetaMarkers (x-axis) or the top 100 adult MetaMarkers (y-axis). (F) Distributions of average preserved developing brain co-expression AUROCs across all genes for organoid and developing brain networks. The redline shows the performance of the adult co-expression network. The scatterplot plots the data in the histogram (y-axis) against the number of cells in each organoid data set (x-axis). The blue line shows performance for a cell down-sampled developing brain data set, with points representing the average performance over 10 random samples and the error bars showing ± 1 standard deviation. (G) Transplanted organoids preserve adult co-expression over developing brain co-expression. [file pbio.3002912.s008.png]

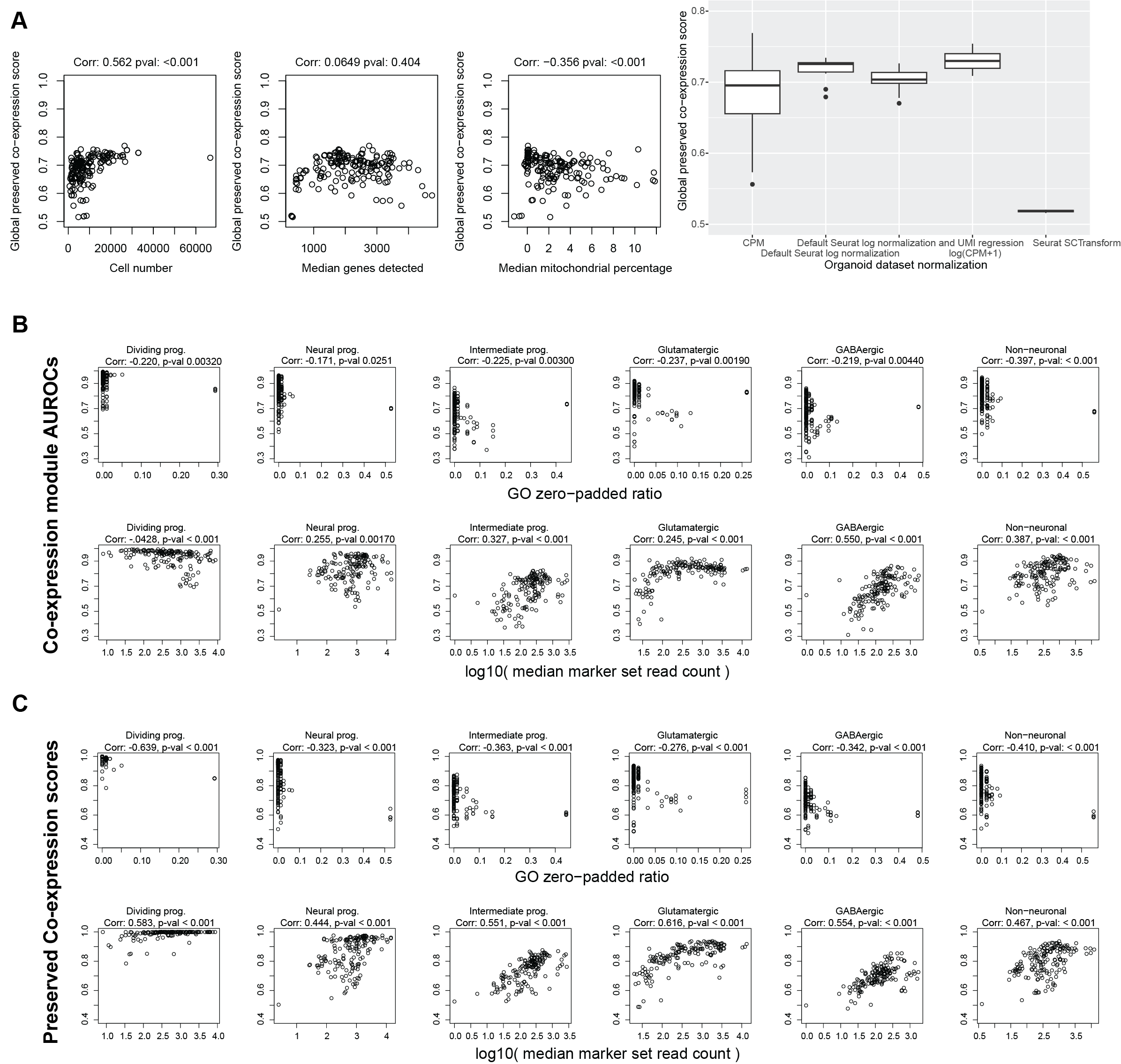

Supplement: S9 Fig — (A) Scatter plots comparing either the cell number, the median number of detected genes, or the median percentage of mitochondrial mapping genes (x-axis) of each organoid scRNA-seq data set to the average preserved co-expression AUROC across all genes (y-axis). The boxplots display the global preserved co-expression score across the RNA-seq normalizations used among the organoid data sets. (B) Scatterplots of either the zero-padded ratio (top row) or marker set expression (bottom row) against the co-expression module scores for each cell type across the organoid data sets. (C) Scatterplots of either the zero-padded ratio (top row) or marker set expression (bottom row) against the preserved co-expression scores for each cell type across the organoid data sets. Underlying data can be found in the Zenodo repository (doi:10.5281/zenodo.13946248). (PNG) [file pbio.3002912.s009.png]

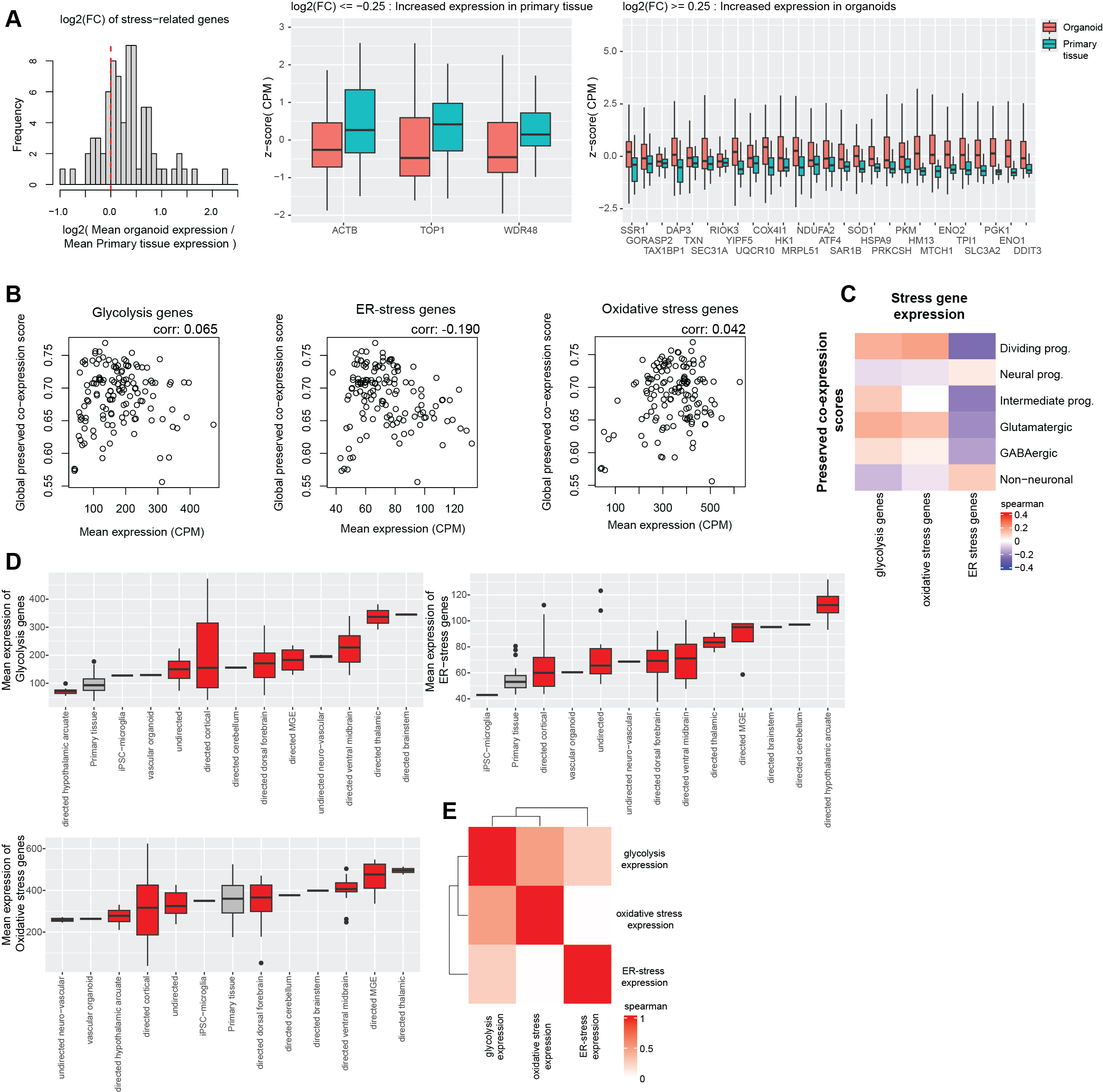

Supplement: S10 Fig — (A) Histogram depicting the log2 fold change (FC) of the 76 stress-related genes as defined in S4 Table, including only genes present within all data set gene annotations. The boxplots compare the expression distributions of the genes with at least a mean expression of 50 CPMs across all data sets and defined as either elevated in expression for primary tissue data sets (log2(FC) < = −0.25, left set of boxplots) or elevated in expression for neural organoids (log2(FC > = 0.25), right set of boxplots). Expression values are the z-scored CPM values for each gene. Only neural organoid and primary tissue data sets that used CPM normalization were used for this analysis. (B) Scatter plots comparing the global preserved co-expression score of all neural organoid datasets against their mean expression (CPM) of either the glycolysis, ER-stress, or oxidative stress genes. (C) Heatmap depicting the Spearman correlations between the mean expression of stress-related gene sets (columns) and the cell type-specific preserved co-expression scores (rows) across the neural organoid data sets that used CPM normalization. (D) Boxplots depicting the distributions of mean expression (CPM) of either the glycolysis, ER-stress, or oxidative stress genes across the neural organoid protocols/data sets and primary tissue data sets that used CPM normalization. (E) Heatmap depicting the Spearman correlations in mean expression (CPM) of the glycolysis, ER-stress, and oxidative stress genes across the neural organoid data sets that used CPM normalization. Underlying data can be found in the Zenodo repository (doi:10.5281/zenodo.13946248). (PNG) [file pbio.3002912.s010.png]
