# Supplementary material for: Meta-analysis of single-cell RNA sequencing co-expression in human neural organoids reveals their high variability in recapitulating primary tissue
Source: PLoS Biol. 2024 Dec 2;22(12):e3002912. doi: 10.1371/journal.pbio.3002912 (PMC11637388; doi:10.1371/journal.pbio.3002912)
Supplement: Attachment — Submitted filename: response_to_reviewers.docx [file pbio.3002912.s015.docx]

We thank the reviewers for their many positive remarks (“assessment is valuable”, “manuscript is quite polished”, “invaluable reference data”, “commendable endeavor”, “interesting, scalable”). Reviewers 1 and 3 noted that they only had minor suggestions, which we believe we have now addressed. As noted by the editor, an important critique is reviewer 2’s observations about data organization. We are keen for other researchers to benefit from the data and assessments we have done and believe we have made significant improvements that address this concern. Reviewer 4 suggests interesting experiments broadly directed toward improving the robustness and interpretability of results. We thought these were valuable suggestions and accordingly have performed new experiments to help clarify our findings. In the below, full reviewer comments are shown in black with our remarks in blue and text from the paper also in quotes.

**Reviewer #1:** Werner and Gillis perform a meta-analysis of neural
organoid gene expression to identify how consistent the signature is.
This is an important endeavor - organoid model systems are a proxy for
a biological system (ie, tissue or tissue-tissue interaction) and
assumed to reflect it with sufficient fidelity. Gene expression,
specifically transcriptional modules, are probably the best way to
answer this since they have been shown to be fairly robust in terms of
multiple data points changing simultaneously, giving additional
statistical power to estimations. Gillis uses MetaMarker, which he
published previously, as part of this assessment and shows that
relatively few genes can robustly predict cell type.

Their sample size is sufficiently powered - millions of cells across
180 scRNAseq datasets total. They compare primary tissue, using
different timepoints and brain regions to identify a core, robust
signal, with organoids. They also show organoids vary in preserving
primary tissue co-expression at the gene and cell-type level, and that
their approach can quantify primary tissue fidelity in organoids. They
flesh out important caveats to the approach and discuss limitations.

The manuscript is quite polished and I don't have any significant
concerns. This type of assessment is valuable for the scRNAseq
community and should be published. Finally, they provide an R package
for the community to compare their own samples, which is very
valuable.

Minor concerns
The figures are blurry. It may be due to the conversion to PDF, but it
should be fixed prior to publication.

We believe the reviewer is right and this was just introduced at some point within the upload system. We will ensure high resolution figures are provided prior to publication.


**Reviewer #2:** Werner and Gillis collected publicly available
single-cell RNA-seq data of human fetal/primary brains and neural
organoids from NCBI GEO and performed meta-analysis of in vivo and in
vitro brain transcriptomics. Surprisingly, they collected over 4
million cells from 181 datasets, which are invaluable reference data
resource for comparison between in vivo and in vitro brain system, and
across different protocol types of organoids. Focusing on gene
co-expression is also interesting topic, since co-expression was not
deeply addressed in previous meta-analyses of scRNA-seq in neural
organoids (Bhadiri et al., Tanaka et al.). However, I feel that the
collected datasets were not well organized, and concerns that their
conclusion is artifact derived from the unorganized datasets. My major
and minor concerns are shown as follow:

We thank the reviewer for their comments and agree that the previous organization of the organoid data can be improved upon. In the revised manuscript, we parse the organoid datasets much more thoroughly (information on specific major concerns included below) and provide additional detailed metadata per dataset in the updated Supplemental Table 1. The additional information includes: GEO series and accession numbers per sample where available, author-described protocols from the datasets’ original publications, a protocol annotation that we provide distinguishing undirected and region-specific directed protocols, as well as the original cited protocol for each study. We also include updated annotations for the age and cell-line of each organoid dataset, as well as associated quality control metrics for all organoid and primary tissue datasets. We excluded 3 prior studies due to previously missed issues with the underlying data and included 6 new studies from recommendations by the reviewers (Fleck 2022, Pellegrini 2020, Vértesy 2022, Bhaduri 2020, Eichmueller 2022, and Kanton 2019).

After re-organizing the organoid datasets, we re-ran all co-expression analysis of the organoid datasets and the results remain largely unchanged. All associated plots in Figures 3 and 4 as well as associated Supplemental Figures have been updated and we provide here comparisons between the previous and current results for the co-expression module scores and preserved co-expression scores (Figure 3D and Figure 4B) of the organoid datasets. We also provide additional analyses in the new Supp. Figures 6, 7, and 9 that more fully explore the variability in our co-expression assessments as they relate to various technical and quality control metrics as well as placing them in reference to primary tissue scores, details included below. Our results and conclusions remain unchanged with the updated organoid dataset organization and our new analyses provide further support for our original observations of decreased and more variable performance across organoid datasets in preserving primary tissue co-expression.

**Figure 3D**

**Previous panel	Current panel**

**Figure 4B**

**Previous panel**

**Current panel**

Major concerns:
*       It is unclear how authors defined 12 protocol types. What is
difference between "neural_induced_blood_vessel" and
"vascularized_cortical"? What is difference between
"dorsal_patterned_forebrain" and "cortical"? Most of cortical
organoids are guided by dorsal patterning. Bhadiri et al. Nature 2020
clearly defined these cortical organoid protocols as least directed,
directed, and most directed. If authors purpose to assess biological
fidelity across different protocols, authors should carefully digest
each organoid protocol, and systematically classify them: guided or
unguided, directed region, stringency of patterning.

In the original manuscript, we defined the organoid protocols as they were described in their own respective publications/datasets. In the revised manuscript, we use a protocol classification that clarifies whether a protocol is undirected or directed as well as the specific-region generated through directed patterning (See bar plots below). One challenge that currently faces the field as a whole is the diversity of protocols in use with little common understood language used to describe them, which also impacts our meta-analysis here. For the example with dorsal_patterned_forebrain versus cortical organoids, the ‘dorsal_patterned_forebrain’ term is used from a single lab to describe their forebrain directed organoids whereas the term ‘cortical organoid’ is used across studies to describe organoids that may contain cortex with a range of regional identities or more patterned identities. The neural_induced_blood_vessel and vascularized_cortical protocols describe two distinct studies with different methods for vascularization, either inducing neural identity in a vascular organoid or co-culturing a neural organoid with endothelial cells, meriting separate protocol descriptions. For our study, it is not our intent to investigate the accuracy of protocol naming schemes and instead is to derive meaningful assessments to primary tissue that is applicable to any organoid dataset. We think our current grouping of organoid protocols is reasonable for this purpose. The author defined protocols and our grouped protocol annotations are provided in the updated Supplemental Table 1. In the updated protocol descriptions, we count 12 distinct protocols (right most bar plot) as well as the iPSC-microglia and vascular organoid (not a neural organoid) protocols, which are included as meaningful outgroups for comparison in our analyses.

*       In Fig. 1B, "vascularized_cortical" has 1 dataset. However,
this is not correct. Shi et al. PLoS Biol 2020 provided total 12
datasets (6 vascularized and 6 non-vascularized cortical organoids). I
think that authors counted some datasets by series accession number
(GSEXXXX) in certain datasets (e.g. Shi et al., Xiang et al.), but
other dataset by sample accession number (GSMXXXX) (e.g. Velasco et
al., Quadrato et al.). The number of dataset should be counted by
biological replicates.

We thank the reviewer for catching this error, the original organization for this dataset was incorrect. We have split the Shi et al 2020 dataset into 4 different datasets: undirected day 65, undirected day 100, vascularized undirected day 65, and vascularized undirected day 100 neural organoids. The vascularized undirected organoids are still top performers for preserved co-expression across cell-types, as seen in the updated Supplemental Figure 5

*       Xiang et al. also provided two different types of organoids
(cortical and MGE). Authors should separate this dataset into two
protocols.

The Xiang et al. 2017 dataset has been properly separated into directed cortical and directed MGE organoids with the updated dataset organization.

*       Is Femando et al dataset for retina-cortical organoid?
According to NCBI GEO, this is a cortical organoid.

The Fernando et al. 2022 dataset was previously incorrectly labeled, the dataset is now correctly labeled as a directed cortical organoid.

*       Why organoid ages are not provided in certain protocols (e.g.
Pollen et al., Xiang et al.)? These datasets also provided organoid
age in NCBI GEO or in the article.

We provide more comprehensive age annotations for the organoid datasets in Supplemental Table 1 after re-visiting each individual publication.

*       Authors collected single-cell transcriptome data, which were
derived from different platforms (e.g. 10X Genomics Chromium,
STRT-seq, Fluidigm C1). Since the number of detected genes varies with
single-cell platform, it is possible that the co-expression patterns
are also affected by the platform difference.

We provide new analyses comparing the performance of organoid datasets to that of primary tissue datasets, while accounting for gene detection rates and cell numbers, in the new Supplemental Figure 7. Briefly, we construct co-expression networks from individual primary tissue brain regions and time points, using the aggregate of the remaining primary tissue data as the reference network to score the preserved co-expression of our cell-type MetaMarker gene sets. These results consistently demonstrate that organoid datasets perform worse and/or more variably than individual primary tissue brain region datasets across gene detection rates and cell numbers, see example plot here. Plots for all 6 of our broad cell-types are in Supp. Figure 7.

*       Improve the summary table of dataset (Supp Table 1). Please
add more valuable information, such as single-cell platform, organoid
type (guided or unguided), and organoid/brain age, donor ID/cell line.
In addition, certain datasets lack GSM accession number. For example,
Shi et al. performed scRNA-seq to vascularized and non-vascularized
organoids with different ages (d65, d100). It is very important which
accession number corresponding to vascularized organoid or normal
organoid.

We provide all of the requested additional metadata in the updated Supplemental Table 1 for the organoid and primary tissue datasets. For some datasets, GSM accession numbers are not available for the individual datasets as we define them. For example, with the Shi et al 2020 vascularized undirected organoids, the entire dataset is made available with the series number GSE131094, but the vascularized/non-vascularized and age information is provided within the barcodes of individual cells, which we manually separated into different datasets.

*       This unorganized dataset may cause unfair batch effect
normalization across datasets, and possibly affect gene co-expression
patterns.

We thank the reviewer for their comments and have re-organized the organoid datasets accordingly. We show our results and conclusions do not change with the new dataset organization and provide additional analyses controlling for technical and quality control metrics, demonstrating the robustness of our original results.

*       Please provide more detail of their cell-type annotations. How
to determine 6 cell types? "Non-neuronal" contains various cell types,
including astrocyte, oligodendrocyte, and microglia, which express
clearly distinguishable markers (e.g. AQP4, MBP, AIF1). In
meta-analysis, I strongly recommend uniformly determining cell types
in each dataset by user-defined rules (e.g. markers) or automatic
annotation software.

For this meta-analysis, we use cell-type annotations as determined by the original studies with the mappings to our 6 broad cell-type annotations provided in the original Supplemental Table 2. We group cell-type annotations at this broad level of classification with our 6 cell-types in an effort to construct a generalizable framework applicable to diverse neural organoid datasets. Certainly, more specific cell-types such as the individual non-neuronal cell-types mentioned can be investigated, a concept we do briefly explore with more specific neuronal sub-types in Figure 4D-E. However, for the purpose of this study, we think our initial broad cell-type annotations are more appropriate for establishing the generalizable nature of our co-expression assessments, with investigations into specific cell-types an excellent starting point for future work.

*       In public data, some low-quality libraries were deposited (in
particular, scRNA-seq library for primary/fetal tissues). Therefore,
these low-quality libraries should be removed before co-expression
analysis. Although authors performed QC with %mitochondria and
detected genes, what percentage of cells were excluded by these
criteria?

We now provide associated quality control metrics for the organoid and primary tissue datasets in the updated Supplemental Table 1, including the median mitochondrial percentage, median feature count, and the number of cells before and after quality control filtering for each dataset. Many of the datasets we collected were made publicly available with quality control filtering already completed, though with variable thresholds across the studies. We re-filtered datasets with uniform quality control thresholds for our analysis, with the percentage of cells filtered per dataset included in the left bar plot below. The vast majority of organoid datasets had less than 10% of cells removed with our thresholds and the dataset with the highest percentage of filtered cells still has ~10,000 quality cells remaining (right bar plot, x-axis order is the same).

*       Trevino et al. dataset was classified as unannotated dataset,
but cell type annotation is provided from NCBI GEO
("multiome_cluster_names.txt.gz")

The provided cell-type annotations for the Trevino dataset are only for the multi-omic data, where for our study we only use the scRNA-seq data, for which there are no cell-type annotations provided.

*       At line 144-147, authors described that they identified sets
of marker genes to define cell types across brain regions. However,
they did not provide any results related to brain regions (Fig. 2 was
performed across timepoint). Although Bhadiri et al. and Braun et al.
performed scRNA-seq in multiple brain regions, whether authors
analyzed them in each brain region separately or merged all brain
regions?

In our original manuscript, Supplemental Figure 2 explored marker set expression across brain regions for the individual primary tissue datasets. We have provided additional cross-regional analysis for the preservation of co-expression in the new Supplemental Figures 6 and 7. Briefly, we construct co-expression networks from individual primary tissue brain regions and time points, using the aggregate of the remaining primary tissue data as the reference network to score the preserved co-expression of our cell-type MetaMarker gene sets. In Supplemental Figure 6, we find that variation in performance across brain regions tracks well with the number of sampled cells per brain region. Supplemental Figure 7 compares the performance of individual primary tissue brain regions to organoid datasets for the preservation of primary tissue co-expression while accounting for cell number and feature detection, as previously described in response to the major concern about performance variability across gene detection rates/platforms.

*       It is interesting that a small number of meta-analytically
derived primary  tissue cell-type markers have high utility in
predicting primary tissue cell-type annotations. However authors must
discuss whether top-ranked MetaMarker gene are consistent with our
previous knowledge. For example, it is expected that GABEergic neurons
display SLC32A1, GAD1, and GAD2 as high-ranked marker, MetaMakrers in
dividing progenitor possibly contain TOP2A and MKI67. If these
well-defined markers are not listed as high rank, authors should
discuss why these genes are not defined as top-ranked meta markers.

We agree that the top ranked MetaMarkers should be made available with this work and provide a new Supplemental Table 3 with the top 20 ranked MetaMarkers for each of our 6 broad cell-type annotations. These markers agree with known biology, the mentioned markers by the reviewer are included in these top 20 MetaMarker lists. The complete lists of 100 ranked MetaMarkers per cell-type are also made available for download through our R package we constructed for this work.

*       Overall, the research strategy is interesting, but each
dataset seems not to be fairly preprocessed (Some dataset combined
multiple protocols, whereas others separated). The current manuscript
remains concern that this unfair preprocessing leads to artifacts of
gene co-expression patterns.
Minor concerns:
*       Provide top-ranked MetaMarkers as supplemental table. This is
very important information in this study.

We thank the reviewer for the opportunity to more accurately organize the organoid datasets and provide evidence that the results from the new dataset organization are largely identical with the original analysis.


**Reviewer #3** (Alessandro Fiorenzano, signs review): In the manuscript
titled "Preservation of Co-expression Defines the Primary Tissue
Fidelity of Human Neural Organoids," the authors present a
meta-analytic pipeline. This approach quantifies gene-level
preservation of primary tissue co-expression, enabling cell-type
specific quantifications within brain organoids. The analysis
encompasses 2.95 million cells from primary brain tissue across 50
datasets, and 1.63 million cells from neural organoids across 130
datasets, each differentiated using distinct patterning protocols.
This extensive examination aims to establish robust cell-type specific
markers and co-expression patterns, facilitating the analysis and
validation of whether in vitro brain organoid models faithfully
replicate authentic molecular identities in culture. Additionally, the
authors offer an R package for the convenient download of the primary
tissue reference co-expression network. This tool facilitates the
analysis of new neural organoid data using straightforward,
meaningful, and efficient statistical methods.

The manuscript introduces novel elements that address a critical
concern in the field of "human biology," particularly within the field
of human brain organoid generation. This issue revolves around the
absence of well-established human standards, presenting a substantial
challenge in the context of advancing 3D modeling (KB Jensen et al.,
Stem Cell Reports 2023). The inherent inaccessibility of the human
brain hampers direct comparisons and validation of results,
potentially leading to the production of unreliable data lacking
essential controls. The authors' efforts to develop a pipeline that
can be utilized across different laboratories may represent an
important tool for validating molecular identity and conducting
quality checks for ongoing in vitro human stem cell brain models. This
commendable endeavour has the potential to significantly contribute to
the standardization and reliability of research in the brain organoid
field.

Only minor points need to be addressed to make the manuscript suitable
for publication.
Please see a list of minor comments below:

1) The manuscript would benefit and provide stronger conclusions by
integrating two relevant studies currently missing in the manuscript:
La Manno et al., Cell, 2016, and Birtele et al., Development 2021. The
authors should include these two studies as they are relevant, using
human fetal mesencephalic tissue to compare hPSC differentiation into
dopamine neurons and midbrain organoids.

We thank the reviewer for the pointer to the suggested datasets. The small size of the suggested fetal datasets (1,977 cells from La Manno et al. 2016 and the 23,483 cells from Birtele et al.) is unlikely to make a measurable impact on our aggregate 3 million primary tissue reference data. At this stage of analysis, each new fetal dataset has a fixed amount of effort, making small datasets somewhat less useful to us; additionally, small datasets may pose additional challenges with respect to standardization when incorporating them into our existing primary tissue reference. We apologize for not satisfying what might seem an easily-accommodated request (and are certainly mindful of making the burden of each new dataset decrease so we can continue to expand our corpus and perform targeted analyses more easily).

2) To facilitate the use of the proposed meta-analytic pipeline, the
authors should provide a detailed list of primary tissue single-cell
datasets in supplementary, including tissue source, the number of
cells analyzed, and quality control. Similarly, the authors should
provide a list of brain organoid datasets, specifying their regional
brain identity and quality control for each dataset analyzed.

We provide an updated Supplemental Table 1 that includes all the requested information for the organoid and primary tissue datasets.

3) While efforts are made to model the human brain, the main datasets
present are derived from the mouse or, at least, from the
differentiation of mouse stem cells, representing a valuable source of
information. It would be helpful for the manuscript if the authors
could discuss the possibility of meta-analytic approaches to study
evolutionary differences and similarities between brain regions of
different species. Additionally, shedding light on how animal models
can be exploited as an information source to decipher aspects of the
human brain would underscore the broad applicability of meta-analytic
approaches.

We agree that this is a profoundly interesting avenue for future research. We are a little apprehensive about even discussing this within the present manuscript, as it is not something we directly assessed within the current work (although this is another project underway in the lab).

**Reviewer #4:** In this paper, Werner and Gillis described their
analytical framework based on marker identification and co-expression
analysis, to estimate fidelity of the molecular programs driving cell
type heterogeneity of primary developing human brains in neural
organoids. The authors demonstrated their approach with unannotated
primary data sets. When applying the analysis to neural organoid
scRNA-seq data, the authors reported generally substantially weaker
preservation, as well as high variability, of cell type marker
co-expression patterns in organoids, suggesting potential limitations
of the current neural organoid protocols.

In general, I think the approach described in the manuscript is
interesting, scalable, and can be indeed applicable to different
systems to derive a general view of system consistency. Meanwhile, I
also have several critical concerns, not necessarily on the approach
itself, but also how much the results obtained with the approach in
comparing neural organoids with primary developing human brains are
informative and useful to the neural organoid communities.

1. My first technical concern is the reliability of the methods
against undesired technical/biological confounding factors. While gene
co-expression is truly a great metric of how close two genes interplay
in biological processes, it can also be affected by many different
technical factors (especially when looking at co-expression on
single-cell level), as well as biological factors which could confound
the interpretation. Technical factors include cell number, data
coverage, etc., and the potentially confounding biological factors
include different cell type composition and proportions in different
data sets, expression levels of different genes and so on. The authors
indeed spent effort to demonstrate that those factors would not affect
the results, which I very much appreciate. Still, they are not yet
convincing enough to me.

  1) For example, the authors show that in primary MetaMarkers per
cell type show the highest average expressions in the expected cell
type generally regardless of brain regions (Suppl. Fig. 2). This,
however, doesn't mean that the co-expression of those genes are of
consistent level in different brain regions. The authors should
directly clarify it, for example, by subseting into different brain
regions and checking the co-expression patterns in different brain
regions are consistent and of high level. In fact, even the showed
results are not perfect (e.g. in many 2nd trimester primary data the
GABA- and glutamatergic neuron markers don't show consistent pattern
in all regions), and that is even just the average of markers instead
of individual markers.

We appreciate the reviewer’s thoughtful remarks on ensuring robustness to technical properties, something we are always happy to address. We provide new analyses performing the suggested experiment of generating region-specific co-expression networks and scoring preserved co-expression. In brief, we construct co-expression networks for individual brain regions in a leave-one-out manner, constructing an aggregate primary tissue reference network from the remaining datasets. The preserved co-expression scores for the top 100 MetaMarkers per cell-type across brain regions are provided in the new Supplemental Figure 6, as well as a plot showing the variation in cell numbers across the individual region datasets. In general, we find that performance variability across brain regions tracks well with variation in cell numbers, suggesting dataset size as a limiting factor. In Supplemental Figure 7, we compare the primary tissue region-specific datasets to our organoid datasets while accounting for cell numbers and gene detection rates. In general, these results demonstrate organoid datasets perform worse and are more variable for preserving primary tissue co-expression compared to individual primary tissue brain regions, while accounting for dataset size and gene detection rates.

An example for the Neural Progenitor scores is provided below, with the scores for all 6 cell types provided in Supplemental Figure 7.

  2) The authors also provided the correlation between zero-padding of
genes (which should be somehow negatively correlated with overall gene
expression and sequencing depth) and preserved co-expression, which I
also appreciate. This analysis, however, focused on how those factors
could affect the gene-set level comparison, and skipped their
potential influence on the overall comparison between data sets. This
should not be very difficult to address, by correlating the overall
co-expression preservation with the technical factors such as cell
number per data set and average detected transcript numbers and so on.

We agree with the reviewer this analysis would be informative to include and provide updated plots in the new Supplemental Figure 9, included below as well. Here, the y-axis is the averaged preserved co-expression AUROC across all genes with the x-axis reporting either the median mitochondrial percentage, median feature count, or cell number for the organoid datasets. The global preserved co-expression score correlates most strongly with the dataset size, while mitochondrial percentage and feature detection are weakly related. We note that the top performing organoid datasets (score >= .7) range quite broadly in cell number.

2. Another technical concern I have is the interpretability of the
method. While the proposed analysis can indeed evaluate the overall
fidelity of a system compared to the reference, it seems to lack of
the capacity to identify potential biological processes which may
contribute to the deficit of the system, which can potentially
enlighten researcher in the field to improve the system. The authors
did explore the possibility by introducing the gene-set level
analysis. I appreciate such effort. Some of the reported findings also
make sense and are interesting, for example, the lack of preservation
on angiogenesis and immune functions. However, this analysis is also
largely limited. As the authors mentioned, the preservation of
co-expression is negatively linked with whether a cell type is
generated or not in the organoid. As non-neural cells including
endothelial cells and immune cells are not expected to be there, both
the lack of co-expression preservation of the non-neural
cell type markers and the reported enriched GO terms don't actually
provide any more insight. Indeed, most researchers in the field care
more about how to make the expected cell types (e.g. neural progenitor
cells, neurons and glial cells) close enough to their primary
counterpart, instead of to make the other non-neuroectoderm-derived
cells altogether. On top of that, as the co-expressions were estimated
with all cells, it is also difficult to tell which cell type are
affected the most.

We agree with the reviewer that many researchers are currently striving to make organoid cell types close enough to primary tissue cell-types, to use the language provided by the reviewer. We think an important step in this direction is simply to obtain quantitative evaluation of how well we are doing. The “why” or “how” is ultimately much more complicated and while the present work may provide some information about that, it isn’t our primary goal. Our intent for this project is similar to efforts for the evaluation of protein structure prediction. The value arises from really strong evaluation, first, which sets the stage for lots of research effort that can iterate based on that feedback. Similarly, the primary goal of the current work is to provide a quantitative measure of fidelity between organoid systems and primary tissue as a means to advance from previous variable and arguably qualitative comparisons. We think our existing and new analyses do substantially advance that goal, addressing a crucial gap in the current literature. We agree that our results showing extremely poor performance for preserving primary tissue co-expression of non-neuronal, vascular, and immune cells is explained by the absence of those cell-types in neural organoids, but we would also like to highlight the large degree of variability in performance we report for the cell-types that are present in neural organoids. These results strongly suggest a fair amount of experimental work remains to produce organoids with high fidelity even at our broad level of cell-type annotations. Our co-expression framework is designed to easily assess new organoid datasets and provides a quantitative means forward for identifying when neural organoids are comparable to primary tissue data, an essential and previously missing tool that can aid in the ongoing effort to produce high fidelity organoids.

3. This study largely focuses on high-level cell type heterogeneity,
which is of course very important. However, the equally, if not more,
important aspect is whether the generated cell types are of the
expected regional and subtype identities. The proposed method and the
reported results don't seem to have the capacity to address this,
which largely limit the value of this study to the neural organoid
community. The authors should explain and clarity.

We agree that the investigation of more specific cell-types is of interest to the community and briefly explore this in Figure 4D-E, comparing the preserved co-expression of specific neuronal sub-type markers for two directed organoid protocols. While generating more specified cell-types within neural organoids is indeed a current ongoing focus for many labs, our results suggest there is still much work to be done in generating comparable cell-types at our broad level of classification. The majority of our organoid datasets are designed to produce excitatory neuronal lineages, yet we document clear deficits and large variability in performance for preserving excitatory co-expression compared to primary tissue data, as one example. It is difficult to see how subtype and regional identities can be produced with high fidelity without first robustly producing comparable cell-types at the broader level of excitatory/inhibitory/etc. However, the breadth of primary tissue data we collected is still informative for investigating more specific cell-types as we briefly explore in Figure 4D-E and is an excellent starting point for future work.

4. So far, there are already quite some studies looking into fidelity
of neural organoids recapitulating developing human brains. Some (e.g.
Camp et al. 2015, Velasco et al. 2019, Uzquiano et al. 2022) focus on
the recapitulation and consistency of neural organoids to primary
development, and others focus on differences between the two systems
(e.g. Bhaduri et al. 2020, Vértesy et al. 2022). There is also a more
recent study in biorxiv (He et al. 2023) which also integrated many
scRNA-seq data of neural organoids and compared with primary
developing human brain atlases similar to this study, but with more
complicated analysis on cell type matching, differential expression
analysis, etc. All those studies somehow highlighted some important
biological processes happened to certain cell types (mostly neural
progenitors and neurons). I don't think this study should provide as
detailed information as the above mentioned studies, given that the
proposed method in this study should
in principle be more ease-to-use and scalable as its pros. Still, I
would expect the authors to refer to and acknowledge those previous
studies, and try to check whether the previous finding can be
confirmed with their method.

We thank the reviewer for the opportunity to expand on how our study compares to contemporaries and have included references to the Vértesy 2022 and He 2023 studies in the updated manuscript, all other mentioned studies were referenced in the original manuscript. A general conclusion from the referenced prior work comparing neural organoids and primary tissue is that neural organoids exhibit elevated expression of stress-related genes, which we now explore in the new Supplemental Figure 10. Below is the additional Results and Discussion text for the new analysis centered on stress gene expression as well as the new Supplemental Figure 10:

**Results lines 659-671:**

“In numerous studies, neural organoids consistently show increased expression of stress-related genes compared to primary tissue(5,12,44,45). We confirm that finding here and examine its correlation with our preserved co-expression scores. While neural organoids exhibit general increased expression of stress-related genes (glycolysis(45), endoplasmic reticulum-stress(5), and oxidative-stress(5)), there is considerable variability among datasets, with many showing levels comparable to primary tissue (Supp. Fig. 10A). Interestingly, stress-related gene expression in neural organoids shows minimal correlation with our preserved co-expression scores at the genome level (Supp. Fig. 10B), with only slight negative correlations between cell-type preserved co-expression and ER-stress expression specifically (Supp. Fig 10C). This suggests that ER-related stress pathways may have an increased impact on cellular identity within neural organoids compared to other stress pathways. Additionally, we observe high variability in stress-related gene expression across different organoid protocols (Supp. Fig. 10 D-E), indicating different protocols may have distinct impacts on cellular stress.”

**Discussion lines 727-738:**

“In neural organoid systems, a consistent finding is the elevated expression of stress-related genes compared to primary tissue, as confirmed in our analysis. However, there are conflicting conclusions regarding the impact of this increased stress-related expression. Some studies report an impairment of cell-type identity among stressed cells(12,44), while others report little effect on core cellular identity programs(38,45). In our study, we observe a slight negative correlation between cell-type-specific preserved co-expression scores and ER-stress-related gene expression, with minimal association found for glycolysis and oxidative stress genes. These findings support the notion that cell stress may compromise cellular identity within neural organoids. Importantly, we note significant variability in stress gene expression across organoid protocols, which may contribute to the discordant observations in previous studies. Overall, extensive experimental work is necessary to investigate the potential relationship between cellular stress and cell-type identity in *in vitro* systems further. ”

**Supplemental Figure 10**

**ER-related stress is lightly related to preserved co-expression within neural organoids**

**A** Histogram depicting the log2 fold change (FC) of the 76 stress-related genes as defined in Supplemental Table 4, including only genes present within all dataset gene annotations. The boxplots compare the expression distributions of the genes with at least a mean expression of 50 CPMs across all datasets and defined as either elevated in expression for primary tissue datasets (log2(FC) <= -0.25, left set of boxplots) or elevated in expression for neural organoids (log2(FC >= 0.25), right set of boxplots). Expression values are the z-scored CPM values for each gene. Only neural organoid and primary tissue datasets that used CPM normalization were used for this analysis. **B** Scatter plots comparing the global preserved co-expression score of all neural organoid datasets against their mean expression (CPM) of either the glycolysis, ER-stress, or oxidative stress genes. **C** Heatmap depicting the spearman correlations between the mean expression of stress-related gene sets (columns) and the cell-type specific preserved co-expression scores (rows) across the neural organoid datasets that used CPM normalization. **D** Boxplots depicting the distributions of mean expression (CPM) of either the glycolysis, ER-stress, or oxidative stress genes across the neural organoid protocols/datasets and primary tissue datasets that used CPM normalization. **E** Heatmap depicting the spearman correlations in mean expression (CPM) of the glycolysis, ER-stress, and oxidative stress genes across the neural organoid datasets that used CPM normalization.

5. The primary reference atlas data sets used in the study are largely
biased to the neocortex and telencephalon, especially the second
trimester data. Many "multi-region" data (e.g. Bhaduri et al.), while
indeed include multiple brain regions, have huge difference on
measured cell numbers in different brain regions. Together with my
first concern, it makes me wonder how representative and robust the
reference co-expression pattern is to cell types in different brain
regions. And regarding the organoid data sets, I also notice that some
high-quality data from well-known labs are not included (e.g.
Pellegrini et al. 2020 from Lancaster lab, Eichmüller et al. 2022 and
Vértesy et al. 2022 from Knoblich lab, Kanton et al. 2019 and Fleck et
al. 2022 from Treutlein lab). I'm therefore curious about the standard
of data selection and whether they are comprehensive and
representative enough.

We thank the reviewer for the suggested organoid datasets and have added them to our analysis, see Supplemental Table 1. Our new region-specific analyses in Supplemental Figures 6 and 7 demonstrate consistent high scores of preserved co-expression across brain regions as well as varying performance that does indeed track with cell numbers across the region-specific datasets. Additionally, when accounting for dataset size, we demonstrate that organoid datasets still perform worse and more variably when compared to the individual brain region datasets (Supplemental Figure 7).

6. A minor concern: I notice that the authors used 'cerebral' in
supplementary table 1 to represent organoids derived using unguided
protocols. This doesn't seem to be the ideal term, as the unguided
protocols are able to generate many non-cerebral, e.g.
non-telencephalic neurons. Using "brain" or "unguided" will be much
more proper, in my opinion.

In general, we describe the organoid protocols as originally done in their associated publications and the term ‘cerebral’ seems to be generally colloquially used to describe unguided neural organoids. In the updated manuscript, we do re-label the protocols to distinguish between undirected and directed protocols. We provide the original authors’ protocol descriptions as well as our grouped annotations in the updated Supplemental Table 1.
