# Supplementary material for: Meta-analysis of single-cell RNA sequencing co-expression in human neural organoids reveals their high variability in recapitulating primary tissue
Source: PLoS Biol. 2024 Dec 2;22(12):e3002912. doi: 10.1371/journal.pbio.3002912 (PMC11637388; doi:10.1371/journal.pbio.3002912)
Supplement: Attachment — Submitted filename: response_to_reviewers.docx [file pbio.3002912.s016.docx]

Reviewer comments:

Reviewer #2: In the revised manuscript, authors re-organize the
collected meta data, provide additional key information, and
re-perform the co-expression analyses. The manuscript is improved, but
still contains several critical concerns. Therefore, before the
publication, these concerns should be revised or addressed by
additional data.
*       Supplementary Table 1 is improved, but it is unclear why Shi
et al. datasets were separated into four groups. This GEO repository
contains 3 biological replicates per group, and these replicates
should be processed separately, since each replicate could not
generate the organoid with the same cell composition and may affect
the co-expression. Is it technically difficult to separate this data
by biological replicate?

We had previously separated the Shi et al. datasets into four groups by age and vascularization status. We have now separated the datasets as requested into separate biological replicates for a total of 12 datasets, as reflected in the updated supplemental table 1.

*       I still have major concerns about their protocol
classification. For example, authors classified Kadoshima et al.
protocol as directed dorsal forebrain, and Eiraku et al. protocol as
directed cortex. Kadoshima's protocol is an improved version of
Eiraku's, and the most of medium compositions are same (See Kadoshima
et al. PNAS, 2013). Esk et al. used Lancaster et al. 2017 protocol.
However, authors classified Esk et al. and Lancaster et al 2017 as
different groups. In addition, Pasca et al. 2015 and Sloan et al. 2018
used the same protocol, but were classified into different groups.
Authors may classify protocols based on the description of the
publications, but the most of publications might not clearly
distinguish "dorsal forebrain" and "cortical".  Although I appreciate
authors' efforts to re-organize the collected datasets, authors should
pay more attention to their protocol classification scheme, since
authors discussed the variability of the "directed cortical"
protocols in the main text (Fig. 3, Supp Fig 4). The interest of the
most of biologists is how the differences of the protocols affect
their fidelity. Since authors submitted the manuscript to a
biology-oriented journal rather than computational biology-related
journals (e.g. PLoS computational biology), the protocol must be
categorized by reasonable and acceptable way for biologists.

We thank the reviewer for their comment on our protocol classification and agree that the primary concern of ensuring datasets are organized appropriately in order to make fair comparisons across datasets is highly important to the interpretability of this paper.

Before describing the new experiments we have performed to provide an integrated classification, we would like to stress that our original reasoning for using author-provided descriptions for classifying protocols is precisely due to the documented fact that studies are variable and inconsistent in how they classify protocols, as excellently summarized in PMID: 36171373, which we now cite. The field as a whole is actively working towards a consensus of reasonable and acceptable guidelines for classifying protocols, which has not yet been achieved.

Related to the above reviewer comment, the reviewer mentions in several of their subsequent comments that protocols should be classified appropriately for their level of direction and that variation in cellular composition across protocols may be driving our co-expression results. Attempting to classify protocols through their respective written methods to determine the expected level of direction and cell-type composition would result in each study receiving its own protocol classification due to the many varied and study-specific methodological choices made throughout our collected datasets.

Instead, we have addressed these comments consistent with our paper’s general approach, by empirically quantifying the cell-type composition of the organoid datasets and then grouping datasets based on their compositional similarities, independent of the protocol classifications. To this end, we have made two substantial changes to this manuscript; we now empirically quantify the cellular composition of the neural organoid datasets (Supp. Fig. 4) and group them by compositional similarities rather than by protocol for our co-expression analyses, and we have removed the vast majority of text and graphs related to data organized by protocol classifications. As a summary of our reasoning, due to the pervasive variability in how organoid protocols are described within the field, we think it provides a more generalizable contribution to empirically categorize them by their cell-type composition to make like-to-like comparisons for our co-expression results. The results and text changes related to specific comments are included further in our response and we have added the following text to the Discussion:

Lines 746-751: “Importantly, we largely avoided direct comparisons of neural organoids across differentiation protocol labels, as naming schemes for protocols are inconsistent across studies and the field as a whole is actively working towards a consensus nomenclature(75), which will require a significant amount of experimental work to fully characterize each protocol. Instead, we grouped datasets empirically through cell-type composition to ensure comparisons were made across similar datasets.”

*       After identification of MetaMarkers, authors addressed "how
well these markers capture organoid temporal and regional (protocol)
variation". Then, authors performed AUROC quantification using top 100
markers from each primary dataset, and obtained consistent performance
regardless of primary dataset or organoid timepoint (except for
neuronal progenitor).  This is reasonable and understandable. In
contrast, to address regional (protocol) variation, authors performed
PCA, and used PC1 weights to assess the variation. This PCA-based
analysis is complicated, and may lead misinterpretation. I think that
more explanation or additional analyses are needed.

We agree this rationale and the results can be clearer. Our central question here is whether our primary tissue MetaMarkers characterize biological variability present across the organoid datasets, which can be directly quantified through PCA. The main observation is that our MetaMarkers are greater sources of expression variability across organoid datasets than non-marker genes, i.e. the MetaMarkers have greater PC1 weights than non-marker genes consistently across organoid datasets. We have clarified and expanded the respective data and text, included below:

Lines 267 – 279:

“**Broad primary tissue cell-type markers characterize organoid expression variability**

Due to the lack of publicly available cell-type annotations for the vast majority of the organoid datasets we sample, we investigate whether our primary tissue MetaMarker gene sets characterize organoid variability through principal component analysis (PCA). Genes that are heavily weighted in the first PC of a transcriptomic dataset are sources of significant variability in gene expression and likely represent important biological variability; this is largely the reasoning for using PCA as a feature selection tool in standard transcriptomic analyses. We find that our lists of 100 primary tissue MetaMarkers have higher PC1 weights (absolute-value of PC1 weights that are then min-max normalized per dataset) than non-marker genes and are consistently heavily weighted in PC1 across organoid datasets more so than non-marker genes (Supp. Fig. 3D). This demonstrates our MetaMarker gene sets characterize greater sources of gene expression variability across organoid datasets compared to non-marker genes.”

Supplemental Figure 3D

**D** MetaMarker and non-marker gene-set distributions of normalized PC1 eigenvalues across all organoid datasets (left plot). The right plot depicts the fraction of datasets each MetaMarker gene (the average for each MetaMarker gene set is reported) is called as ‘heavily weighted’ in PC1 for a given normalized eigenvalue threshold (x-axis).

-       For example, PC1 weights are highly variable among organoid
protocols (Supp. Fig. 3C). Even within directed dorsal forebrain
protocols, PC1 weights are quite different. According to their new
Supplemental Table 1, the dorsal forebrain protocol is composed of
four original protocols, Kadoshima et al, Sloan et al., Qian et al.
and Esk et al. Is this due to the difference of original protocols?

We think there may have been a miscommunication around the intent of the PC analysis. The question we are seeking to answer here is do the metamarkers capture a lot of the expression variability within an experiment, regardless of experiment. We find that MetaMarkers are consistently more weighted in PC1, indicating they do measure a lot of the total expression variability. However, we are not suggesting that total expression variability (magnitude of PC1, say) is itself a useful measure. We think that would be overly broad and influenced by many other factors (and we discern no obvious pattern in the data). We have edited the text to try to make our intent here clearer (also discussed a bit in the next question).

-       Is PC1 really good assessment measurement for
protocol/regional variation? Instead of PCA, how about using top100
markers per primary region as did in Supp. Fig. 3B and Supp. Fig 2?
From new Supplemental Table 1, primary tissues data seems to contain
the corresponding regions to certain organoid protocols (e.g.
ganglionic eminence, thalamus, hypothalamus). Authors identifies top
100 markers for one primary region, and calculate AUROC in the
organoid protocols for this region, other regions, and undirected
organoid protocols.

-       If the suggested method is technically difficult, please
carefully explain the rationale of PCA-based analysis.

We think we poorly explained this in the original text. We have tried to clarify the purpose of this modest analysis. We wanted to assess whether the markers were capturing broad expression variability. We wanted to do this in a way that was independent of cell-type annotations since the vast majority of the organoid datasets we use do not have cell-type annotations publicly available.

Note that this is a distinct question from analyses we performed focused on cell-type annotation in the primary tissue datasets. There is some overlap in the sense that they both ask “are the metamarkers important” but in the primary tissue datasets we can answer that with a cross-validation approach on the markers, which we believe the reviewer is suggesting we apply to the organoids as well. But we can’t comprehensively take this approach because we don’t have cell-type annotations to validate against. So, PCA is a less precise and less useful answer – but a more general one.

-       A word "capture" in the section title is very vague…. Does
author claim that broad primary tissue cell-type markers are
independent from organoid temporal/regional (protocol) variation?

We have changed the word to ‘characterize’, which we think is a better description of our stated goal for this analysis; quantifying whether our primary tissue MetaMarkers represent biological variability across organoid datasets.

*       MetaMarkers for non-neuronal cell types seem to be biased to
endothelial cells and microglia (RAB31, C1QC, C1QB, C1QA, and R2PY12)
(Supp Table 3). Therefore, co-expression module AUROC is very high in
vascular organoid, neuro-vascular, iPSC-microglia and vascularized
undirected organoid. In addition, undirected protocol also can
generate mesodermal progenitors (precursor of endothelial cells and
microglia) (Ormel et al., Nat Commun 2018), whereas directed protocols
cannot. Since many scientists imagine "non-neuronal"  cell type as
oligodendrocyte or astrocyte, this may lead misinterpretation. Authors
should discuss this as limitation of their cell type classification
approach in the manuscript, or carefully classify "non-neuronal" cells
into each glial cell type.

We thank the reviewer for this comment and have adjusted the primary tissue cell-type annotations as pointed out by the reviewer, excluding microglia from the non-neuronal classification. In the revised manuscript, only oligodendrocytes, OPCs, and astrocytes are included in the non-neuronal cell-type annotation. We have redone all the analyses in this paper with these updated primary tissue cell-type annotations; computing new MetaMarkers and redoing the primary tissue cross-validation, as well as re-doing all co-expression analyses with the updated MetaMarkers. Supplemental table 3 now includes MetaMarkers for the Microglia/Immune cell-type annotation, which include P2RY12, C1QC, C1QB, and C1QA as noted by the reviewer. The top 20 Non-neuronal MetaMarkers now include recognizable markers for astrocytes, oligodendrocytes, and OPCs (BCAN, SCRG1, S100B, NKX2-2, OLIG2, PLP1, PMP2). Excluding microglia from the Non-neuronal MetaMarkers did have a significant impact on our co-expression results for the Non-neuronal MetaMarkers throughout the manuscript; in general, performance in organoids for non-neuronal co-expression increased substantially. Below, we include a comparison of the preserved co-expression scores for all 7 broad cell-type annotations. It is clear that the exceptionally poor previous performance for non-neuronal co-expression in organoids was driven by the inclusion of microglia within the non-neuronal cell-type annotation. While performance for non-neuronal co-expression in organoids has improved, organoid datasets still generally fail to produce preserved co-expression scores comparable to primary tissue.

This is the same plot as in Figure 4B, but includes the Microglia/Immune preserved co-expression scores.

*       Line 360: "Interestingly, the protocol with the most variable
performance for cell-type co-expression across datasets is the
directed cortical protocol, which can be classified as an intermediate
protocol in terms of directed differentiation between the undirected
and region-specific protocols". Is this true? Usually, the direction
level of the organoid is controlled by signaling inhibitors or ligands
(Bhadiri et al., Nature 2020). Have authors interpreted directed
cortical protocols as "intermediate protocols" by the differences of
signaling inhibitors/ligands. If so, please more carefully explain why
the directed cortical protocols can be examined as "intermediate"?
Authors categorized five different protocols (Field et al. 2019,
Popova et al., 2021, Revah et al., 2022, Vertesy et al., 2022, Xiang
et al., 2017, two authors-developed, Lancaster 2017-based,
Eiraku-based, and Pasca-based) into directed cortical. Is it possible
that the variability may be due to the
variation of protocols rather than the direction level.

As above, we have removed text comparing results related to protocol classifications and now group organoid datasets by their shared cellular composition. Our original rationale for the use of ‘intermediate protocol’ was based on the general descriptions of the level of protocol direction within previous studies, where directed cortical protocols would be between undirected and region-specific protocols in terms of the level of direction. We recognize this is an imprecise way to describe protocols and have removed this text from the manuscript, instead focusing on comparing organoid datasets through cellular composition.

*       Line 370: "While variability in co-expression performance may
reflect compositional differences among neural organoids, the dramatic
range in performance across the directed cortical organoid datasets
reveals extensive variability in fidelity to primary tissue."  & Line
433: "regardless of putative compositional variation across organoid
protocols and/or treatments, we demonstrate the vast majority of
organoids from all sampled protocols consistently fail to preserve
glutamatergic or GABAergic primary tissue co-expression at a level
comparable to primary tissue". For these conclusions, their results
are insufficient. As also discussed in the manuscript, the AUROC may
be affected by the compositional variation of cell types. Since the
dorsal cortical organoid group is a mixture of multiple protocols, it
is possible that the cell composition is highly different among
protocols compared to other groups. To support their conclusion,
authors should provide data showing that the
AUROC is not or weakly dependent on the cell composition (e.g.
correlation between cell type ratio and AUROC, as did in Supp Fig. 9)
or tone down this conclusion.

We think this raises an excellent point and agree with the reviewer that cellular composition may vary substantially across the labeled protocol types and we now directly quantify and group datasets by cellular composition instead of by protocol label (Supp. Fig. 4A-C). Briefly, we assign cell-type annotations in organoids based on enriched expression of the top 25 MetaMarkers per cell-type. Comparing this approach to author provided cell-type annotations in the temporal directed dorsal forebrain datasets shows very high agreement in annotations (Supp. Fig. 4A), with some missannotations between non-neurons and neural progenitors (Supp. Fig. 4B), most likely due to some shared expression of neural progenitor MetaMarkers within astrocytes, that we document in Supp. Fig. 1B. As a summary of our results based on cellular composition, included below, we find that organoid datasets composed of very small percentages of a cell-type (0-10%) produce both co-expression models scores (Supp. Fig. 4D) and preserved co-expression scores (Supp. Fig. 5C) that match datasets composed of larger percentages (ranging from ~10-90% across cell-types). This is most clear for the glutamatergic, GABAergic, non-neuronal, and intermediate progenitor co-expression, where neural progenitor co-expression scores decrease with larger neural progenitor percentages, most likely due to missannotations between neural progenitors and astrocytes (Supp. Fig. 4B and D, Supp. Fig. 5 C). These results demonstrate that organoid datasets produce comparable co-expression scores largely independent from their cell-type percentage; additionally, the majority of datasets with near zero co-expression signal (~0.5) are the datasets within the 0-10% bins, likely the datasets that do not produce the respective cell-type. In summary, co-expression performance in organoids is largely determined by whether or not the given cell-type is present and is not dependent on the exact amount of the cell-type present.

Our new analyses show our co-expression scores are largely uninfluenced by the underlying variation in cellular composition across neural organoids. With that in mind, organoids still do not produce preserved co-expression scores that are comparable to primary tissue datasets for nearly all the 6 broad cell-types. There is virtually no overlap in the preserved co-expression scores between primary tissue and neural organoids for non-neuronal, intermediate progenitor, and GABAergic MetaMarkers, while a small subset of organoid datasets does produce comparable scores for neural progenitor and glutamatergic MetaMarkers (Fig 4B). Dividing progenitor scores are nearly identical. We think our conclusions match our results appropriately now that we show our co-expression results are not impacted by compositional variation across organoid datasets. The updated text and relevant figures are included below.

Lines 319-357: “The variation among individual organoid datasets for co-expression of the primary tissue cell-type markers may be influenced by the compositional variation in cell-types produced across the differentiation protocols. A protocol that aims to produce a directed excitatory lineage organoid is not expected to produce inhibitory cell-types and thus should not necessarily exhibit strong inhibitory neuron co-expression. We test this relationship by first estimating the cell-type compositions of each neural organoid dataset through their expression of the primary tissue MetaMarkers. Briefly, cells are scored for their aggregate expression of genes within each MetaMarker gene set (using the top 25 MetaMarkers per cell-type) and then assigned a cell-type label determined by the MetaMarker gene set with the highest enriched expression. Comparing this annotation approach to author-provided annotations for the temporal directed forebrain neural organoid atlas reveals high concordance in cell-type annotations (Supp. Fig. 4A), with some missannotations between neural progenitors and non-neurons (Supp. Fig. 4B), again most likely attributable to the shared expression of the neural progenitor MetaMarkers with astrocytes. We then group neural organoid datasets by their shared cell-type percentages and compare their co-expression module scores, revealing pervasively weak relationships between a dataset’s cell-type percentage and its associated co-expression module score, particularly for the differentiated cell-types (Supp. Fig. 4C-D).

Starting with glutamatergic composition, the range of co-expression module scores for datasets with 0-10% glutamatergic cells is 0.399-0.920, which spans from no signal (~0.5) to signal comparable to primary tissue scores (~>0.85, Supp. Fig. 4D). Organoid datasets with greater than 10% glutamatergic cells (spanning > 10% to 90%) have scores ranging from 0.560-0.911, exceedingly similar to datasets with 10% or less glutamatergic cells. This demonstrates organoids with small glutamatergic percentages (< 10%) are producing glutamatergic co-expression relationships equal to datasets with much higher glutamatergic percentages (10-90%). The GABAergic, non-neuronal, and intermediate progenitor cell-type compositions display the same trend when comparing the lowest cell-type percentage datasets to all other datasets (GABAergic: [0-10%] scores 0.312-0.826, (10-90%] scores 0.440-0.862. Non-neuronal: [0-10%] scores 0.497-0.929, (10-60%] scores 0.683-0.948. Intermediate Progenitors: [0-5%] scores 0.379-0.826, (5%-20%] scores 0.371-0.822. Supp. Fig. 4D). The neural and dividing progenitors are the exception, with higher estimated cell-type percentages trending with lower co-expression module scores, which is more apparent with the neural progenitors than the dividing progenitors (Supp. Fig. 4D). This is most likely due to the missannotations between neural progenitors and non-neurons (likely astrocytes). In general, the majority of organoid datasets with near-zero co-expression signal are also the datasets with near-zero predicted composition of the respective cell-type, particularly for the differentiated cell-types (Supp. Fig 4D). This suggests co-expression performance within neural organoids is influenced largely on whether or not the cell-type is present within the organoid, regardless of the exact prevalence of the cell-type.”

Lines 456-477: “In contrast, organoid datasets vary substantially in preserved co-expression scores across our primary tissue MetaMarker gene sets, with exceedingly small overlaps in scores to primary tissue datasets for all MetaMarker gene sets except dividing progenitors (Fig. 4B). As before with our quantification of intra-gene set co-expression, compositional variation across organoid protocols may influence the variation in performance across cell-types/datasets. We again compare the preserved co-expression scores across datasets grouped by cell-type composition and find the same trend as before with the intra-marker set co-expression module scores; organoids ranging from small to large cell-type percentages produce largely similar preserved co-expression scores (Supp. Fig. 5C). We quantify variability in preserved co-expression across neural organoids in a slightly different manner by computing the correlation in preserved co-expression between all MetaMarker gene sets, revealing significant positive correlations across all cell-type marker sets in neural organoids (spearman correlations range from 0.426-0.886, FDR-adjusted p-values are all < .001, Fig. 4C). This indicates preserved primary tissue co-expression is a global feature of organoid datasets, which is not expected if variability in cell-type composition among neural organoids was a strong determinant of preserved co-expression. Importantly, regardless of the cell-type composition of neural organoids, we demonstrate the vast majority of the sampled organoids consistently fail to preserve primary tissue co-expression at a level comparable to primary tissue for all the broad cell-types except dividing progenitors (Fig 4B, exceedingly small overlap in the primary tissue and organoid distributions). This suggests a persistent remaining deficit in the fidelity of neural organoids in reference to primary tissue that is independent of variability in cell-type composition.”

**Supplemental Figure 4: Intra-marker set MetaMarker co-expression is weakly related to cell-type composition**

**A** Scatter plots comparing the cell-type annotation percentage of individual organoid datasets from the annotated directed dorsal forebrain temporal datasets, with author-provided annotations on the x-axis and annotations determined by MetaMarker expression on the y-axis. **B** Confusion matrix for the results in A, comparing the MetaMarker predicted annotations to the author provided annotations. **C** Heatmap displaying the predicted cell-type percentages (rows) of all the organoid datasets (columns), hierarchically clustered by the organoid protocol. The plot to the left of the heatmap depicts the distributions of unassigned cell percentages per dataset, where large percentages of unassigned cells are dependent on the expression normalization used in individual studies. Studies with those normalizations were excluded from Supp. Fig. 4D and Supp. Fig. 5C. **D** Boxplot distributions comparing the predicted cell-type percentage (top boxplot plot per cell-type, binned in intervals of 10-percentage points) to the co-expression module score (bottom boxplot plot per cell-type) for all neural organoid datasets. The x-axes are the same for the top and bottom sets of boxplots per cell-type.

Supplemental Figure 4

Supplemental Figure 5C**:** Boxplot distributions comparing the predicted cell-type percentage (top boxplot plot per cell-type, binned in intervals of 10-percentage points) to the preserved co-expression score (bottom boxplot plot per cell-type) for all neural organoid datasets. The x-axes are the same for the top and bottom sets of boxplots per cell-type.

*       Line 438: "Organoids across protocols additionally exhibit
near-zero preservation of non-neuronal primary tissue co-expression,
suggesting neural organoids generally do not produce or produce
extremely dysregulated non-neuronal cell-types (Fig. 4B, Supp. Fig.
5)."  Due to the strong bias of non-neuronal cell types to endothelial
cells and microglia, no preservation of non-neuronal primary tissue
co-expression is due to lack of these cell types in the organoid, but
not dysregulation of non-neuronal cell types.

The non-neuronal cell-type annotation and MetaMarkers now reflect astrocyte and oligodendrocyte biology. While the performance across organoid datasets for non-neuronal co-expression has increased compared to our previous results, organoid datasets still do not achieve preserved co-expression of primary tissue non-neuronal markers that is comparable to primary tissue datasets, as evidenced by the almost complete lack of overlap in the non-neuronal distributions between primary tissue and organoid datasets in Figure 4B.

*       Section: "Fidelity of finer resolution cell-types through
preserved co-expression". This section is very interesting, but hard
to read for biologists. Especially, "finer resolution cell type" may
be difficult to be understood by biologists. How about changing it to
"region-specific cell types"?

We agree region-specific cell-type is a more accurate description and have edited the corresponding text.

*       Line 541: "These results highlight preserved primary tissue
co-expression of ECM-related genes as a particularly consistent
deficit across neural organoids, suggesting that investigations into
the signaling between artificial ECM and cells in organoid cultures
may be a route forward for general improvements of organoid fidelity."
ECM is one of major characteristics of endothelial cells. ECM genes
are highly expressed in endothelial cells, but not in other neuronal
cell types. Therefore, the poor conservation of ECM genes in organoid
is due to the lack of endothelial cells.

To test this hypothesis, we now repeat this analysis with primary tissue co-expression networks derived using only the 6 broad neural lineage cell-types, which would exclude endothelial cells. We find the same ECM-related GO terms are the top ranked terms by p-value (Supp. Fig. 8C), supporting our original interpretation. There are 6 of these ECM genes that are shared between the original and now modified experiments, which we list in the manuscript.

*       In the temporal variation analysis of the preserved
co-expression (Fig. 5), did authors use top 100 or top 10 cell-type
markers? Authors described top 100 in main text, whereas Fig 5A
described top 10.

The preserved co-expression score (y-axis) in Figure 5B is the average AUROC for the top 100 MetaMarkers per cell-type as described in the text. We have removed the text in the Figure 5A graphic, which was describing the top 10 co-expressed partners of an individual gene used to compute an individual AUROC.

*       Line 566: "Our approach in predicting organoid annotations in
Figure 2 is based on aggregate marker expression and did not produce
temporally variable results, whereas our approach here comparing
preserved co-expression of the same marker genes does produce
temporally variable results.".  Authors selected MetaMarkers as
markers in broad primary tissue cell types among time points and brain
regions (Fig. 2), whereas the analyses in Fig. 5 used markers in each
primary dataset. Therefore, there is no surprising about this
conclusion. Rather than comparing with Fig. 2, authors should compare
the results of Fig. 5 with Supp. Fig. 3. If my understanding is
correct, Fig. 5 was assessed by the preservation of co-expression,
while Supp. Fig. 3 was evaluated by gene expression levels to define
cell type annotation. This is well matched with their final conclusion
"This indicates that the co-expression relationships of genes rather
than their expression levels better capture temporal
variation in developing systems".

There was a typo in the previous manuscript, we were indeed referencing Supp. Fig. 3 rather than Figure 2. The corresponding text has now been edited.

*       I think that the relationship of temporal variation with
co-expression and expression levels is very different among cell
types. For example, neuronal progenitors showed the temporal variation
in both Fig. 5 and Supp Fig. 3. In contrast, intermediate progenitors,
GABAergic neurons, and glutamatergic neurons showed temporal variation
in the co-expression preservation, but not in cell type annotation. I
think that this is interesting, and authors should discuss these
differences among cell types in main text.

We think the temporal variation in marker expression in neural organoids is actually fairly weak and we do note in the text that younger primary tissue markers have sudden jumps in performance, suggesting the trend is more likely due to dataset intersections of technical variability. The temporal trend in preserved co-expression is apparent across all cell-types and so is less likely to be driven purely by technical variation. In that respect, what we conclude in the results is that temporal variation in co-expression among the cell-types is actually very similar, as they all appear to switch around GW9-10.

*       For the comparison with Supp Fig. 3, it is better to add the
co-expression analysis for dividing progenitors and non-neuronal cell
type in Fig. 5.

The revised Figure 5 now includes all 6 cell-types.
